# Gallium Mesoporphyrin IX-Mediated Photodestruction: A Pharmacological Trojan Horse Strategy To Eliminate Multidrug-Resistant *Staphylococcus aureus*

**DOI:** 10.1021/acs.molpharmaceut.1c00993

**Published:** 2022-04-13

**Authors:** Klaudia Michalska, Michał Rychłowski, Martyna Krupińska, Grzegorz Szewczyk, Tadeusz Sarna, Joanna Nakonieczna

**Affiliations:** † Laboratory of Photobiology and Molecular Diagnostics, Intercollegiate Faculty of Biotechnology, University of Gdansk and Medical University of Gdansk, Abrahama 58, Gdansk 80-307, Poland; ‡ Laboratory of Virus Molecular Biology, Intercollegiate Faculty of Biotechnology, University of Gdansk and Medical University of Gdansk, Abrahama 58, Gdansk 80-307, Poland; § Department of Biophysics, Faculty of Biochemistry, Biophysics and Biotechnology, Jagiellonian University, Gronostajowa 7, Krakow 30-387, Poland

**Keywords:** isd system, photodynamic therapy, porphyrins, targeted delivery, MRSA

## Abstract

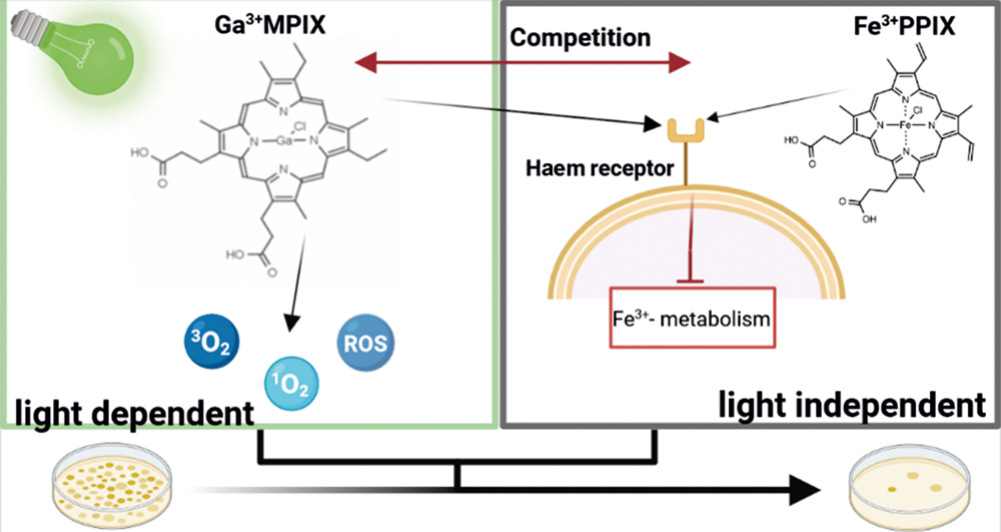

One of the factors determining efficient antimicrobial photodynamic inactivation (aPDI) is the accumulation of a light-activated compound, namely, a photosensitizer (PS). Targeted PS recognition is the approach based on the interaction between the membrane receptor on the bacterial surface and the PS, whereas the compound is efficiently accumulated by the same mechanism as the natural ligand. In this study, we showed that gallium mesoporphyrin IX (Ga^3+^MPIX) provided dual functionality—iron metabolism disruption and PS properties in aPDI. Ga^3+^MPIX induced efficient (>5log_10_ reduction in CFU/mL) bacterial photodestruction with excitation in the area of Q band absorption with relatively low eukaryotic cytotoxicity and phototoxicity. The Ga^3+^MPIX is recognized by the same systems as haem by the iron-regulated surface determinant (Isd). However, the impairment in the ATPase of the haem detoxification efflux pump was the most sensitive to the Ga^3+^MPIX-mediated aPDI phenotype. This indicates that changes within the metalloporphyrin structure (vinyl vs ethyl groups) did not significantly alter the properties of recognition of the compound but influenced its biophysical properties.

## Introduction

In 1928, Alexander Fleming discovered penicillin, which revolutionized medicine and improved the quality of human life. Currently, after almost 100 years, one of the main challenges for both the academic and pharmaceutical industries is antibiotic multidrug resistance (AMR). According to the report of O’Neil, 10 million deaths per year would be caused by AMR infections by 2050.^1^ Antimicrobial photodynamic inactivation (aPDI), primarily used to photokill cancer cells,^2−4^ is now considered an alternative method for eradication of both Gram-positive and Gram-negative bacteria with different drug response profiles.^5−7^ The aPDI approach is based on three components: oxygen, light, which activates a dye known as a photosensitizer (PS). In an oxygen-rich environment, reactive oxygen species (ROS) might be generated through either energy (type II mechanism) or electron (type I mechanism) transfer from an irradiated PS. ROS generated in aPDI are cytotoxic because of their multitarget action on proteins, lipids, or nucleic acids. The ideal PS should exhibit low dark toxicity and high phototoxicity, which usually correlates with a high quantum yield of ROS photogeneration and application safety toward eukaryotic cells. Photodynamic inactivation eradicates microbial species efficiently despite their drug resistance profile.^8−10^ Moreover, recent studies by Woźniak et al. revealed a synergy between photodynamic therapy and clinically used antimicrobials.^11,12^ aPDI has an impact on the production of virulence factors, which cause pathogens to be less virulent.^13,14^ Despite our recent studies of aPDI tolerance and an increased stress response upon consecutive cycles of sublethal treatments,^15,16^ resistance to photodestruction has not yet been observed. aPDI is efficient in both in vitro^17^ and in vivo studies.^18,19^

**Figure 1:**
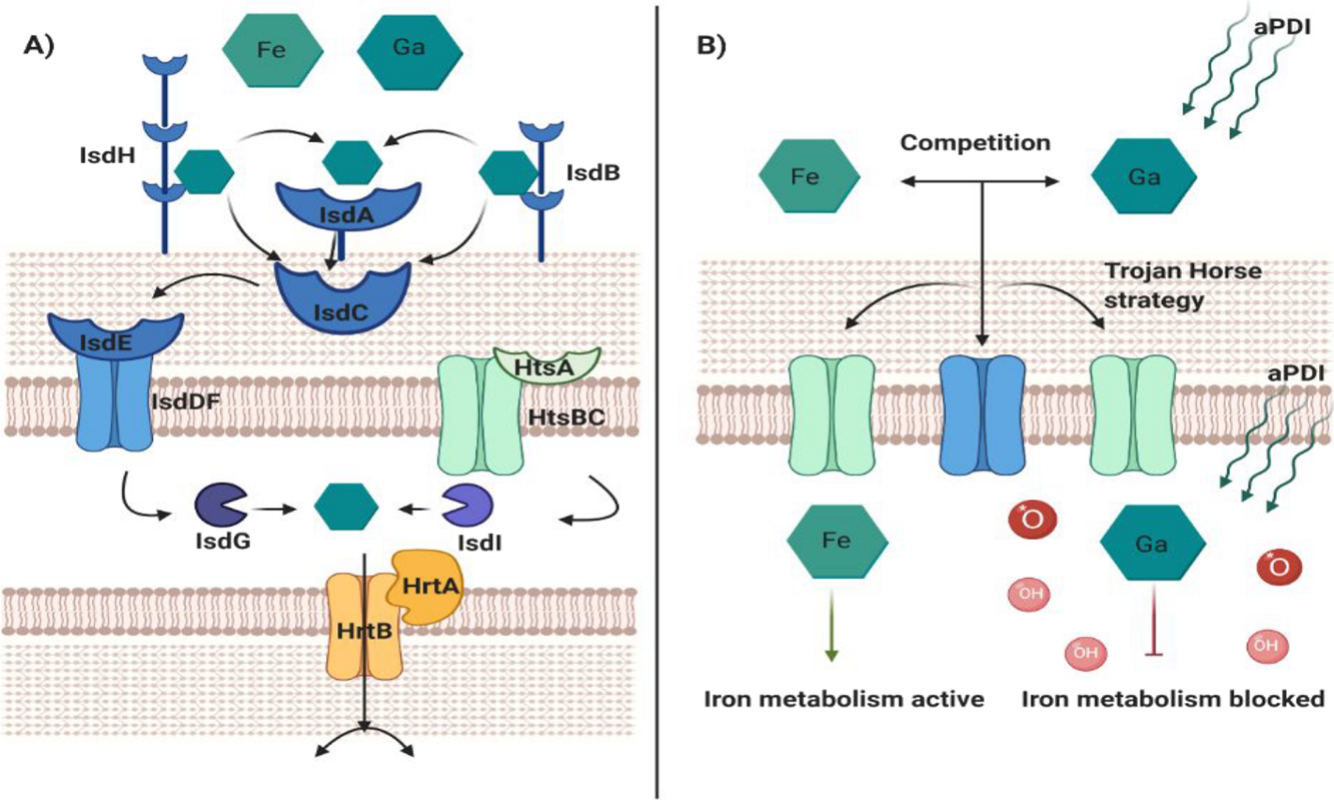
Haem acquisition machinery in *S. aureus* and proposed uptake of gallium^3+^ porphyrin conjugates via the Trojan Horse strategy. (A) In *S. aureus*, Fe^3+^protoporphyrin IX (known as haem) is recognized by Isd and Hts protein machineries. Haem complexed with hemoglobin or haptoglobin–hemoglobin is recognized and released by IsdB and IsdH cell wall-anchored receptors. Also, free haem in the environment is bound to IsdA and then transferred through the cell wall to membranes by IsdCDEF. Another uptake mechanism is known as HtsABC, which directly transfers haem from the cell wall to the cytoplasm. Haem oxygenases (IsdG and IsdI) recognize and cleave the porphyrin structure. In the higher level of haem, the HrtAB detoxification machinery is upregulated and acts as an efflux pump. (B) Because of the structural similarity between Fe^3+^protoporphyrin IX and gallium MPs, bacteria do not distinguish compounds and could take up gallium conjugates. Gallium MPs might work as “Trojan Horse” and disrupt bacterial cells by blocking iron metabolism. In addition, because of the porphyrin ring structure, those compounds generate ROS including singlet oxygen upon light exposure. (Created with BioRender)

The efficiency of aPDI might be dependent on PS uptake.^20,21^ PSs can accumulate in a different manner, depending on the wall structure of bacterial cells, environmental factors, and the type of the involved mechanism, for instance, active transport.^22^ The concept of targeted PS recognition is based on PS uptake using membrane receptors, which recognize PSs as a similarly structured natural ligand. Proposed compounds for targeted PSs are metals conjugated with a protoporphyrin (metalloporphyrins, MPs).^23^ The gallium protoporphyrin IX and gallium mesoporphyrin IX conjugates, formed with the metal oxidation state III, mimic the haem structure (Fe^3+^ protoporphyrin IX) and thus possibly bind to elements of haem acquisition machinery.^24,25^ Gallium compounds are active in disturbing iron metabolism by intracellularly accumulating via the Trojan Horse strategy (Figure 1B).^23,26^ Previous studies showed that Ga^3+^PPIX displayed light-independent antimicrobial activity against both Gram-positive and Gram-negative bacteria by blocking iron metabolism.^26−31^ Gallium MPs also demonstrated antibiofilm activity.^32,33^ Moreover, Ga^3+^PPIX exhibited antimicrobial photodynamic action against *Staphylococcus aureus*.^34,35^

*S. aureus* is a Gram-positive member of ESKAPE pathogens that can effectively “escape” antibacterial drug action.^36^ During infection, the pool of available iron is limited to pathogens such as *S. aureus*. To overcome the low iron availability, bacteria assimilate haem in either free form or bound in complexes with hemoglobin or haptoglobin in vivo (Figure 1A).^37^ The classified mechanism of the *iron-regulated surface determinants* (Isd) or *haem transport system* (Hts) for acquiring iron ions from haem has been reviewed in detail previously.^37,38^ Briefly, IsdH and IsdB are primary receptors for haptoglobin–hemoglobin complexes or hemoglobin alone, respectively.^39^ Both contain conserved *near transporter* domains that recognize and extract haem from the complex.^40^ IsdA protein binds haem from the environment or receives the compound from IsdH or IsdB membrane receptors. Then, haem is transferred through the cell wall to the IsdC component and to IsdE, a membrane lipoprotein. IsdE acquires the haem and delivers it to IsdF, an ATP-binding cassette (ABC) permease. By ATP-hydrolyzing energy produced by IsdD, haem is passed through the membrane to the cytoplasm, where IsdG and IsdI, haem oxygenases, release iron from the structure of haem.^37,39,41,42^ The second well-known iron assimilation machinery is the membrane-localized ABC transporter HtsABC. Hts works in a similar manner to the complex of IsdDEF. HtsA is a membrane-associated lipoprotein, while HtsBC are two ABC transporters. However, their role is described mostly in the transport of staphyloferrin A.^38,41^

Paradoxically, haem itself may induce toxicity at higher concentrations.^43^ The two-component *haem-regulated transporter* (HrtAB) detects and pumps an overdose of the compound out of the cell. HrtAB is an ABC-type transporter, where HrtA acts as an ATPase and HrtB is a permease with the role of a membrane transport channel.^37,39,41^ Deletion of genes encoding the HrtAB transporter revealed an impairment of bacterial growth under high concentrations of haem.^44^ Efflux pump gene expression is regulated by the *haem sensor system* (HssRS), which is required for the adaptive response to haem.^45^

Based on the existing knowledge concerning haem transport in *S. aureus*, we investigated whether Ga^3+^ mesoporphyrin IX (Ga^3+^MPIX) could accumulate and act as a PS against methicillin-resistant *Staphylococcus aureus*. To answer these questions, we evaluated the antimicrobial effect using Ga^3+^MPIX against several staphylococcal strains, including clinical isolates with the multidrug resistance (MDR) phenotype and haem acquisition mutants. Our hypothesis assumed that Ga^3+^MPIX can be efficiently accumulated or retained in *S. aureus* because of the presence or absence of specific haem transporters. In addition, the intracellular activity of Ga^3+^MPIX can act in two ways: independent of light (blocking haem metabolism) or dependent on light (photodynamic action).

## Experimental Section

### Bacterial Strains and Culture Media

This study was conducted with several *S. aureus* strains listed in Table 1.

**Table 1:** Staphylococcal Strains Used in This Study

*S. aureus* strain	relevant characteristic(s)	source/reference
ATTC 25923	reference strain	ATCC
4046/13	MDR strain (Table S1)	clinical blood isolate
1814/06	MDR strain (Table S1)	clinical blood isolate
Newman NCTC 8178	wild-type (WT) strain	
ΔHtsA	ΔhtsA via allelic replacement	(38)
ΔHrtA	ΔhrtA via allelic replacement	(44)
ΔIsdD	*isd::erm*	(37)
5 N	*sec^+^, tsst-1^+^,* spa type: *t2223,* MSSA strain	clinical nasal isolate from adult patients with atopic dermatitis

Bacterial cultures were grown for 16–20 h with shaking (150 rpm) in either the iron-rich medium tryptic soy broth (TSB) (Biomerieux, France) or iron-deficient TSB treated with Chelex-100 (Sigma–Aldrich, USA) and supplemented with 400 μM MgSO_4_. The *S. aureus* ΔIsdD strain was cultured in the presence of erythromycin 10 μg mL^–1^ (Fluka, Buchs, Switzerland).

### Chemicals

**Figure 2:**
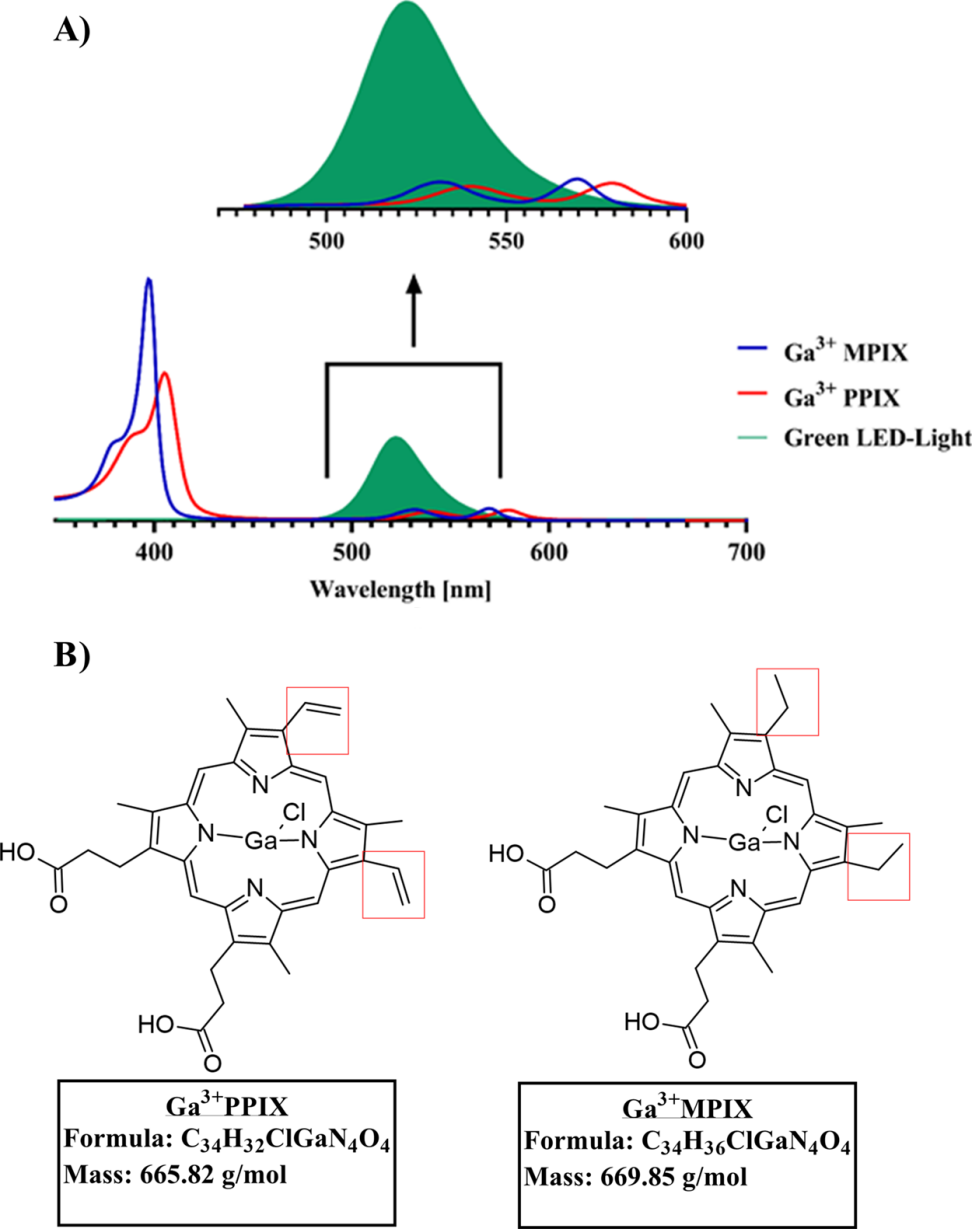
Characteristic of Ga^3+^PPIX and Ga^3+^MPIX. (A) Absorbance spectra of Ga^3+^PPIX and Ga^3+^MPIX titrated to PBS buffer with the imposed scheme of the emission spectrum of a green LED light source used in this study (λ_max_ = 522 nm, FWHM = 34 nm). (B) Ga^3+^PPIX and Ga^3+^MPIX chemical structure, created in ChemSketch. Graphics created in GraphPad Prism 9.

Ga^3+^ mesoporphyrin IX chloride (Ga^3+^MPIX) (Figure 2B) and Ga^3+^ protoporphyrin IX chloride (Ga^3+^PPIX) (Figure 2B) were purchased from Frontier Scientific, USA; stock solutions were prepared according to manufacturer recommendations and kept in the dark at 4 °C. Ga^3+^MPIX was dissolved in 0.1 M NaOH to 1 mM concentration, whereas 1 mM stock of Ga^3+^PPIX was diluted in the mixture 50:50 (v:v) 0.1 M NaOH:DMSO Protoporphyrin IX (PPIX) was purchased from Sigma–Aldrich, USA (Figure S2A); a 1 mM solution was prepared in dimethyl sulfoxide (DMSO) and stored in the dark at room temperature. Protoporphyrin diarginate (PPIXArg_2_, Figure S2B), delivered by the Institute of Optoelectronics, Military University of Technology, Poland, was dissolved in Milli-Q water and stored at −20 °C in darkness until use.^10^ Haem (Sigma–Aldrich, USA) was dissolved in 0.1 M NaOH solution and kept in the dark at 4 °C.

### Light Source

Illumination was performed with a light-emitting diode (LED) light source, emitting green light (λ_max_ = 522 nm, irradiance = 10.6 mW/cm^2^, FWHM = 34 nm, Figure 2A) (Cezos, Poland).

### Photoinactivation Experiments

Microbial cultures were grown overnight in medium in the absence or presence of iron ion concentrations. Then, cultures were adjusted to an optical density of 0.5 McFarland units (McF) (approx 10^7^ CFU/mL) and transferred to a 96-well plate alone or with PS combined. The aPDI samples treated with Ga^3+^MPIX were incubated at 37 °C with shaking in the dark for 10 min and illuminated with different green light doses up to 31.8 J/cm^2^. The number of colony-forming units (CFU/mL) was determined by serial dilutions of 10 μL aliquots and plating bacterial cells on TSA plates. The control consisted of untreated bacteria. TSA plates were incubated at 37 °C for 16–20 h, and then CFU/mL were counted. Lethal and sublethal aPDI conditions were defined similarly to our previous studies.^15,18^ For competition testing, Ga^3+^MPIX was mixed with different concentrations of haem in a mixture with a volume ratio of 1:1 (v/v) and then incubated and irradiated as in photoinactivation experiments. Molar ratios of the studied molecules were as follows: (Ga^3+^MPIX:haem—μM:μM) 1:0, 1:1, 1:10, and 0:10. Each experiment was performed in three independent biological replicates.

### Growth Curve Analysis

Overnight culture was diluted 1:20 (v/v) in TSB or TSB-Chelex medium. A chosen PS (Ga^3+^MPIX, Ga^3+^PPIX, PPIX, or PPIXArg_2_) was added to 450 μL aliquots of bacterial strain to a final concentration of 10 μM. The control group of bacterial cells was not treated with any PS. Prepared samples were loaded into 48-well plates and then placed in an EnVision Multilabel Plate Reader (PerkinElmer, USA), where the optical density (λ = 600 nm) was measured every 30 min for 16 h with incubation at 37 °C with shaking (150 rpm).

### Time-Resolved Detection of Singlet Oxygen Phosphorescence

A solution of the PSs in D_2_O-based phosphate buffer containing a small amount of DMSO (pD adjusted to 7.8) in a 1 cm fluorescence cuvette (QA-1000; Hellma, Mullheim, Germany) was excited for 15 s with laser pulses at 532 nm, generated by an integrated nanosecond DSS Nd:YAG laser system equipped with a narrow-bandwidth optical parameter oscillator (NT242-1k-SH/SFG; Ekspla, Vilnius, Lithuania), operating at 1 kHz repetition rate. The near-infrared luminescence was measured perpendicularly to the excitation beam using a system described elsewhere.^46^ At the excitation wavelength, the absorption of Ga^3+^MPIX was 0.196, while that of Ga^3+^PPIX was 0.235. The measurements were typically carried out in air-saturated solutions. To confirm the singlet oxygen nature of the detected phosphorescence, measurements were compared at 1215, 1270, and 1355 nm by employing additional dichroic narrow-band filters NBP, (NDC Infrared Engineering Ltd., Bates Road, Maldon, Essex, UK) and in the presence and absence of 5 mM sodium azide, a known quencher of singlet oxygen. Quantum yields of singlet oxygen photogeneration by the PSs were determined by comparative measurements of the initial intensities of 1270 nm phosphorescence induced by photoexcitation of rose bengal and the PSs with 532 nm laser pulses of increasing energies, using neutral density filters. The absorption of rose bengal solution, used as a standard of singlet oxygen photogeneration, was adjusted to match that of the examined PSs.

### Electron Paramagnetic Resonance (EPR) Spin Trapping Measurements

EPR spin trapping was carried out using 100 mM 5,5-dimethyl-1-pyrroline N-oxide (DMPO) (Dojindo Kumamoto, Japan DMPO) as a spin trap. Samples containing DMPO and about 0.1 mM of the PSs in 70% DMSO/water with pH adjusted to neutral pH were placed in 0.3 mm-thick quartz EPR flat cells and irradiated in situ in a resonant cavity with green light (516–586 nm, 45 mW cm^–2^) derived from a 300 W high-pressure compact arc xenon illuminator (Cermax, PE300CE-13FM/Module300W; PerkinElmer Opto-electronics, GmbH, Wiesbaden, Germany) equipped with a water filter, a heat reflecting mirror, a cut-off filter blocking light below 390 nm, and a green additive dichroic filter 585FD62-25 (Andover Corporation, Salem, NC, USA). The EPR measurements were carried out employing a Bruker-EMX AA spectrometer (Bruker BioSpin, Germany), using the following apparatus settings: 10.6 mW microwave power, 0.05 mT modulation amplitude, 332.4 mT center field, 8 mT scan field, and 84 s scan time. Simulations of EPR spectra were performed with EasySpin toolbox for Matlab.^47^

### MTT Survival Assay

HaCaT cells (CLS 300493) were seeded at a density of 1 × 10^4^ cells per well in 96-well plates 24 h before the experiment. Cells were divided into two plates for light and dark treatment. Cells were grown in a standard humidified incubator at 37 °C in a 5% CO_2_ atmosphere in Dulbecco’s modified Eagle’s medium (DMEM). Ga^3+^MPIX was added to a final concentration of 0–100 μM and then incubated for 10 min at 37 °C in the dark. HaCaT cells were washed twice with PBS and covered with fresh PS-free DMEM. Next, the cells were illuminated with 522 nm light (dose: 31.8 J/cm^2^). Twenty-four hours post-treatment, MTT reagent [3-(4,5-dimethylthiazol-2-yl)-2,5-diphenyltetrazolium bromide] was added to the cells, and the assay was conducted.^17^ The results are presented as a fraction of untreated cells and calculated as the mean of three independent biological experiments with the standard deviation of the mean. The data were analyzed using two-way analysis of variance (ANOVA) and Tukey’s multiple comparisons test in GraphPad software. A *p* value <0.05 indicated a significant difference.

### Analysis of Real-Time Cell Growth Dynamics

HaCaT cells (CLS 300493) were seeded the day before treatment in seven technical replicates for each condition at a density of 1 × 10^4^ per well on E-plate PET plates (ACEA Biosciences Inc., USA). Cells were grown in a standard humidified incubator at 37 °C and in a 5% CO_2_ atmosphere in DMEM in the xCELLigence real-time cell analysis (RTCA) device (ACEA Biosciences Inc., USA).^17^ When cells were estimated to be in the exponential phase of growth (cell index (CI) = ∼2), the experiment was conducted. The PS was added to the cells at a concentration of 0, 1, or 10 μM and left for 10 min in the dark incubation at 37 °C. Then, the cells were washed twice with PBS, and the medium was changed to PS-free. Afterward, light-treated cells were exposed to 522 nm light (dose of light: 31.8 J/cm^2^). In the case of dark-treated cells, plates were incubated for the corresponding time of irradiation in the dark at room temperature. Then, the plates were returned to the xCELLigence device, and the cell index was measured every 10 min and recorded automatically until the cells reached the plateau phase under each condition.

### PS Accumulation

Microbial overnight *S. aureus* cultures were adjusted to an optical density of 0.5 McF. Ga^3+^MPIX was added to 800 μL bacterial aliquots to final concentrations in the range of 1–10 μM. In a competition assay, bacterial cultures were cultivated in TSB-Chelex medium and incubated with a mixture of PS and haem to obtain an appropriate ratio of concentrations. Samples were incubated for 10 min or 2 h at 37 °C in darkness with shaking. After incubation, the bacterial cells were washed twice with PBS, and lysates were prepared by incubating in a solution containing 0.1 M NaOH/1% sodium dodecyl sulfate (SDS) (w/v) for 24 h at room temperature to lyse cells. The fluorescence intensity of 100 μL of each sample was measured in the dark in 96-well plates spectrophotometrically with the use of an EnVision Multilabel Plate Reader (PerkinElmer, USA). Intracellular accumulation of the PS was calculated based on its calibration curve prepared in lysis solution. Uptake values are presented as PS molecules accumulated per cell based on the accumulation protocol and the following formula.^48^
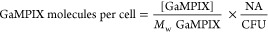
where [GaMPIX] is the concentration [g/mL] of molecules obtained from a calibration curve based on known concentrations of the compound, *M*_w_ is the molecular weight of GaMPIX 669.85 g/mol), NA is the Avogadro’s number (6.023 × 10^23^), and CFU is the colony-forming unit obtained using serial dilutions counted for 1 mL of the analyzed samples.

### Confocal Microscopy Imaging

*S. aureus* Newman and its isogenic ΔHrtA, ΔHtsA, and ΔIsdD mutants were grown in either iron-rich or iron-poor medium overnight for 16–20 h. Then, microbial cultures were diluted to an optical density of 0.5 MacF units. Cells were incubated with 10 μM Ga^3+^MPIX for 2 h at 37 °C with shaking. In control cells, tested compounds were not added. Bacterial samples were washed once in PBS buffer. Afterward, cells were imaged using a Leica SP8X confocal laser scanning microscope with a 100× oil immersion lens with excitation at 405 nm and fluorescence emission at 551–701 nm (Leica, Germany).

### Statistical Analysis

Statistical analysis was performed using GraphPad Prism 9 (GraphPad Software, Inc., CA, USA). Quantitative variables were characterized by the arithmetic mean and the standard deviation of the mean. Data were analyzed using two-way ANOVA and Tukey’s multiple comparison test. A *p* value of <0.05 indicated a significant difference.

## Results

### Gallium MPs Delayed Staphylococcal Growth Light-Independently

**Figure 3:**
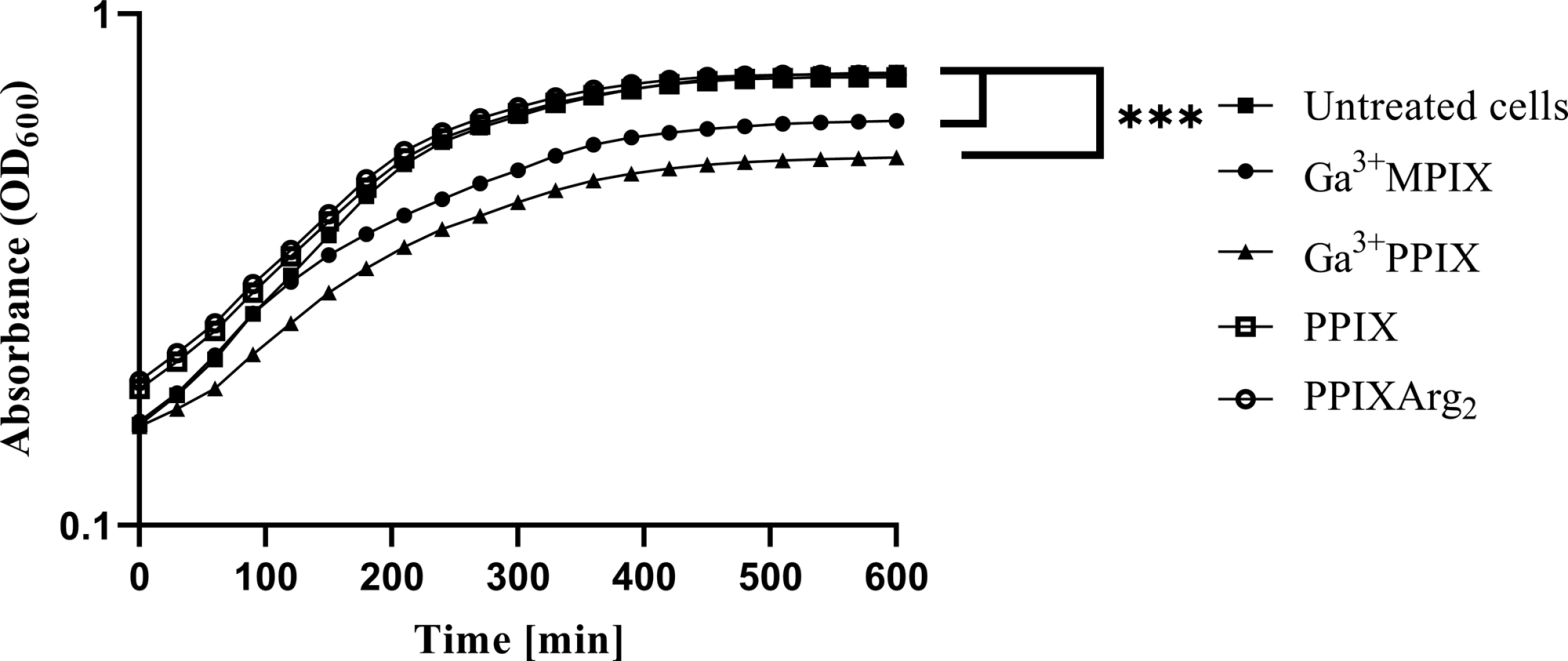
Staphylococcal growth under exposure to porphyrin compounds. Overnight cultures of the *S. aureus* 25923 reference strain in TSB medium were diluted 1:20 (v:v) and exposed to 10 μM Ga^3+^MPIX, Ga^3+^PPIX, PPIX, or PPIXArg_2_. The growth of each condition was monitored by measuring the optical density at 600 nm (OD_600_) on an Envision plate reader. The experiment was conducted in three independent biological repetitions. Significance at the respective *p*-values is marked with asterisks (***p < 0.001) with respect to untreated *S. aureus* 25923 cells.

Previous studies on several MPs have revealed the broad spectrum of gallium ion toxicity by blocking iron metabolism.^23^ We assumed that the presence of ethyl groups in the macrocycle structure of Ga^3+^MPIX (instead of vinyl groups in Ga^3+^PPIX) would not affect its toxicity. The growth of the *S. aureus* 25923 reference strain was compared after exposing cells to gallium MP (Ga^3+^PPIX, Ga^3+^MPIX) and non-MP (PPIX and PPIXArg_2_) in incubation during constant cultivation in iron-rich medium (Figure 3, Table S2). A slower specific growth rate (μ_max_) at the exponential phase was observed after exposure of the *S. aureus* 25923 strain to Ga^3+^MPIX (μ_max_ = 0.15) or Ga^3+^PPIX (μ_max_ = 0.126) compared to untreated cells (μ_max_ = 0.354). Exposure to non-MPs such as PPIX (μ_max_ = 0.282) or water-soluble PPIXArg_2_ (μ_max_ = 0.282) did not influence *S. aureus* 25923 growth. These observations confirm that despite the difference in the structure (ethyl groups vs vinyl groups), Ga^3+^MPIX still induces dark toxicity against *S. aureus*, which is related to the presence of gallium ions in the compound.

### Green-Light Irradiation of Ga^3+^MPIX Generates ROS

To check the mechanism underlying the photodynamic potential of Ga^3+^MPIX, direct measurements of the PS ability to photogenerate ROS were performed.

**Figure 4:**
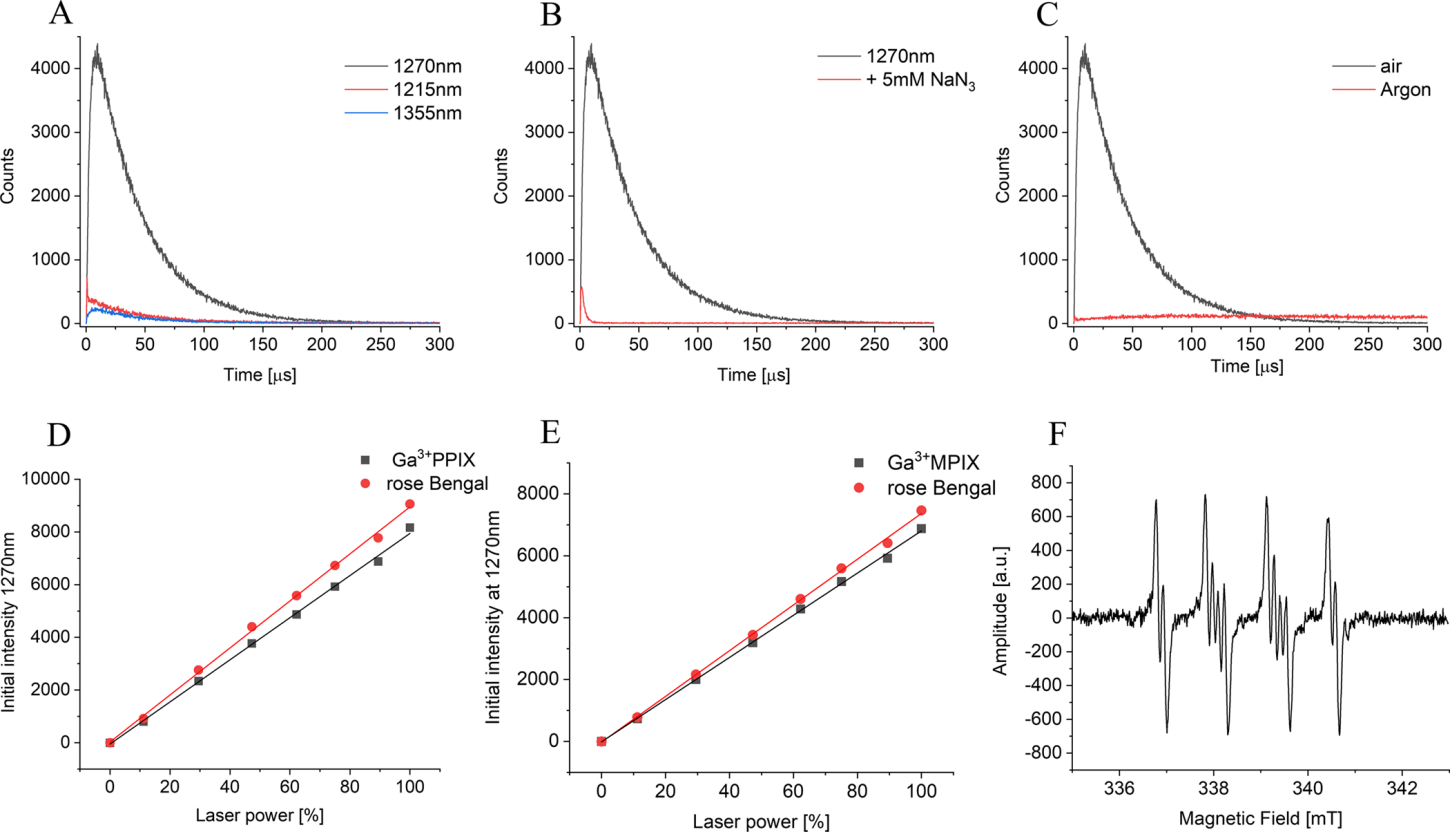
Direct detection of Ga^3+^MPIX- and Ga^3+^PPIX-generated ROS. Photoreactivity of Ga^3+^MPIX and Ga^3+^PPIX showing signals with 1270 nm wavelength, characteristic of singlet oxygen phosphorescence (A), with sodium azide, singlet oxygen quencher (B), and argon-saturated sample (C). Quantum yields of singlet oxygen photogeneration compared to rose bengal (D, E). Samples were irradiated with 532 nm laser light. Characteristic superoxide/DMPO adduct can also be observed with EPR spin trapping (F), although the signal was of low intensity.

Excitation of the PSs by 532 nm laser pulses induced phosphorescence that was strongly dependent on the observable wavelength (Figure 4A). Thus, intense phosphorescence was only observed at 1270 nm, which coincides with maximum emission of singlet oxygen in water. Although D_2_O phosphate buffer was used, the apparent lifetime of the observed phosphorescence was about 40 μs, which is shorter than that reported in pure D_2_O. This shortening could be attributed to a small amount of DMSO and H_2_O, which were used to prepare stock solutions of the PSs. Consistent with the singlet oxygen nature, the observed phosphorescence was significantly quenched by the addition of 5 mM azide (Figure 4B). The quencher reduced both the intensity and lifetime of the phosphorescence, which is most likely due to the quencher interaction with the triplet excited state of the PS and quenching of singlet oxygen. The final test of singlet oxygen nature of the 1270 nm phosphorescence is the effect of exchanging air for argon in the examined samples (Figure 4C). It is evident that saturating the PS solutions with argon completely abolished the singlet oxygen phosphorescence. A weak long-lasting phosphorescence detected in argon-saturated samples could be attributed to emissive relaxation of the porphyrin triplet excite states.

Quantum yields of singlet oxygen photogeneration of the examined PSs, employing rose bengal as a standard for singlet oxygen photogeneration with a yield of 0.75,^49^ were determined to be very similar for both dyes −0.69 for Ga^3+^MPIX and 0.67 for Ga^3+^PPIX, indicating that in aqueous media these porphyrin derivatives are efficient photogenerators of singlet oxygen (Figure 4D,E).

Using EPR spin trapping, we were able to detect, after irradiation with green light (516–586 nm) of the PSs in a mixture of DMSO/H_2_O, the spin adduct with spectral parameters consistent with that of DMPO-OOH, (AN = 1.327±0.008 mT; AH*α* = 1.058±0.006 mT; AH*β* = 0.131±0.004 mT^50^) indicating the photogeneration of superoxide anions (Figure 4F). While both PSs photogenerated, under the conditions used, a superoxide anion, Ga^3+^PPIX was a slightly more efficient generator of the oxygen radical. However, it must be stressed that the yield of generation of superoxide anions by the examined PSs is rather low and cannot be compared with their ability to photogenerate singlet oxygen.

The production of ROS in vitro has also been confirmed by the use of ROS detection probes (HPF and SOSG) after irradiation with two light doses, 12.72 and 31.8 J/cm^2^, in the presence of Ga^3+^MPIX at two concentrations (Supplementary Figure S3A,B). We could observe quite a good correlation with the lower concentration of the compound used (1 μM). In both tested ROS types (HPF for radical detection and SOSG for singlet oxygen detection), we could observe that at a higher light dose (31.8 J/cm^2^) the signals for both probes were higher compared to the lower dose (12.72 J/cm^2^). However, at a higher (10 μM) Ga^3+^MPIX concentration, this relationship is completely lost, which indicates that this is the maximum signal that can be obtained under our experimental conditions. Both ROS are generated during aPDI with Ga^3+^MPIX, which confirms the photodynamic properties of this compound.

In addition, using ROS quenchers, we examined the predominant types of ROS produced during aPDI in vivo, which are likely responsible for the observed death of bacterial cells. We used quenchers of free radicals predominantly formed by type I photochemistry (mannitol, superoxide dismutase), singlet oxygen generated by type II photochemistry (NaN_3_), and ROS formed by mixed type I/II photochemistry (tryptophan, Trp). We observed cell protection after the use of the type II quencher NaN_3_ and Trp, indicating that singlet oxygen was mainly responsible for cell death (Figure S3C). Interestingly, the enzyme catalase (CAT) also caused a statistically significant protection of bacterial cells against Ga^3+^MPIX, which indicates the potential role of H_2_O_2_ in the Ga^3+^MPIX-mediated cell death process. On the contrary, mannitol, superoxide dismutase (SOD) did not provide significant protection. This is in agreement with the small amounts of photogenerated superoxide anions detected by EPR trapping. In summary, singlet oxygen appears to be the major ROS produced during Ga^3+^MPIX-mediated aPDI in vitro and in vivo, and the amount of singlet oxygen produced is comparable to Ga^3+^PPIX.

### Phototreatment of *S. aureus* with Ga^3+^MPIX Reduced Bacterial Viability despite Divergent MDR Profiles

Based on its absorbance spectrum, Ga^3+^MPIX might be excited by green LED light because of peaks called Q-bands, which are near the emission spectrum of the light source used (Figure 2A). However, the aPDI efficiency might differ among strains of *S. aureus*.^9^ Several staphylococcal strains with divergent MDR profiles and different origins were taken for further investigations with 10 μM Ga^3+^MPIX or Ga^3+^PPIX illuminated with green light. The results are presented in Table 2 as the means of viability reduction for a discriminating light dose of 31.8 J/cm^2^. A reduction of more than 3 log_10_ units in the number of CFUs (99.9%) was considered a bacterial eradication/lethal dose. However, sublethal doses were defined as a 0.5–2 log_10_ reduction in CFU/mL.^14^ Light-only treatment and light-independent, 50 min exposure to 10 μM of each compound did not influence the bacterial viability. Ga^3+^MPIX revealed a higher efficiency in bacterial reduction upon green illumination than Ga^3+^PPIX. For Ga^3+^PPIX-mediated aPDI, only sublethal conditions were obtained, despite the 5 N strain, where sublethal reduction was not even reached. Ga^3+^MPIX-mediated aPDI resulted in bacterial eradication for strains: ATCC 25923, clinical isolate 5 N, and 4046/13. In the case of MDR strain 1814/06, aPDI with the Ga^3+^MPIX compound reduced bacterial viability, achieving lethal doses. The response to Ga^3+^MPIX-mediated aPDI is strain-dependent, however, independent of the MDR profile. Interestingly, the addition of a single wash step to the aPDI protocol influenced the effectiveness of aPDI, albeit in different ways (Supplementary Table S3). We observed that the inclusion of a single wash step in the photoinactivation protocol resulted in a better performance of Ga^3+^PPIX-mediated aPDI against bacteria. With Ga^3+^MPIX, the results were more diffuse, some strains were less efficiently photoinactivated, and others remained unchanged or increased. Nevertheless, the efficacy of Ga^3+^MPIX was still better than that of Ga^3+^PPIX. Because of the higher efficiency of Ga^3+^MPIX-mediated phototreatment under green light illumination, we chose this compound for further analysis.

**Table 2:** Phototreatment of *S. aureus* Strains with Ga^3+^PPIX or Ga^3+^MPIX with Green LED Light

strain	mean reduction of survival (log_10_ CFU/mL)^a^ ± SD				
	Ga^3+^MPIX		Ga^3+^PPIX		light-only
	light (+)	light (−)	light (+)	light (−)	
25923	5.33 ± 0.065****	0.022 ± 0.03	1.11 ± 0.65*	–0.03 ± 0.08	0.21 ± 0.28
4046/13	4.1 ± 0.5***	0.05 ± 0.07	1.01 ± 0.15**	0.04 ± 0.05	0.00 ± 0.03
1814/06	2.9 ± 0.24***	0.07 ± 0.11	1.83 ± 0.03***	0.22 ± 0.08	0.15 ± 0.16
5 N	6.08 ± 0.1****	–0.03 ± 0.16	0.34 ± 0.082	–0.07 ± 0.1	0.077 ± 0.09

a Phototreatment conditions: 10 min preincubation with 10 μM Ga^3+^PPIX or Ga^3+^MPIX at 37 °C with shaking without washing; green LED light 31.8 J/cm^2^; log_10_ CFU/mL reduction was assessed with respect to nontreated cells, initial number of cells ∼10^7^ CFU/mL. Light (+)—light-dependent; Light (−)—light-independent; light-only—bacterial cells irradiated without any PS applied. Significance at the respective *p*-values marked with asterisks: * = *p* < 0.05, ** = *p* < 0.01, *** = *p* < 0.001, and **** = *p* < 0.0001 with respect to “Light-only” treatment.

### Phototreatment of *S. aureus* with Ga^3+^MPIX Effectively Reduced Bacterial Viability in an Fe-Dependent Manner

**Figure 5:**
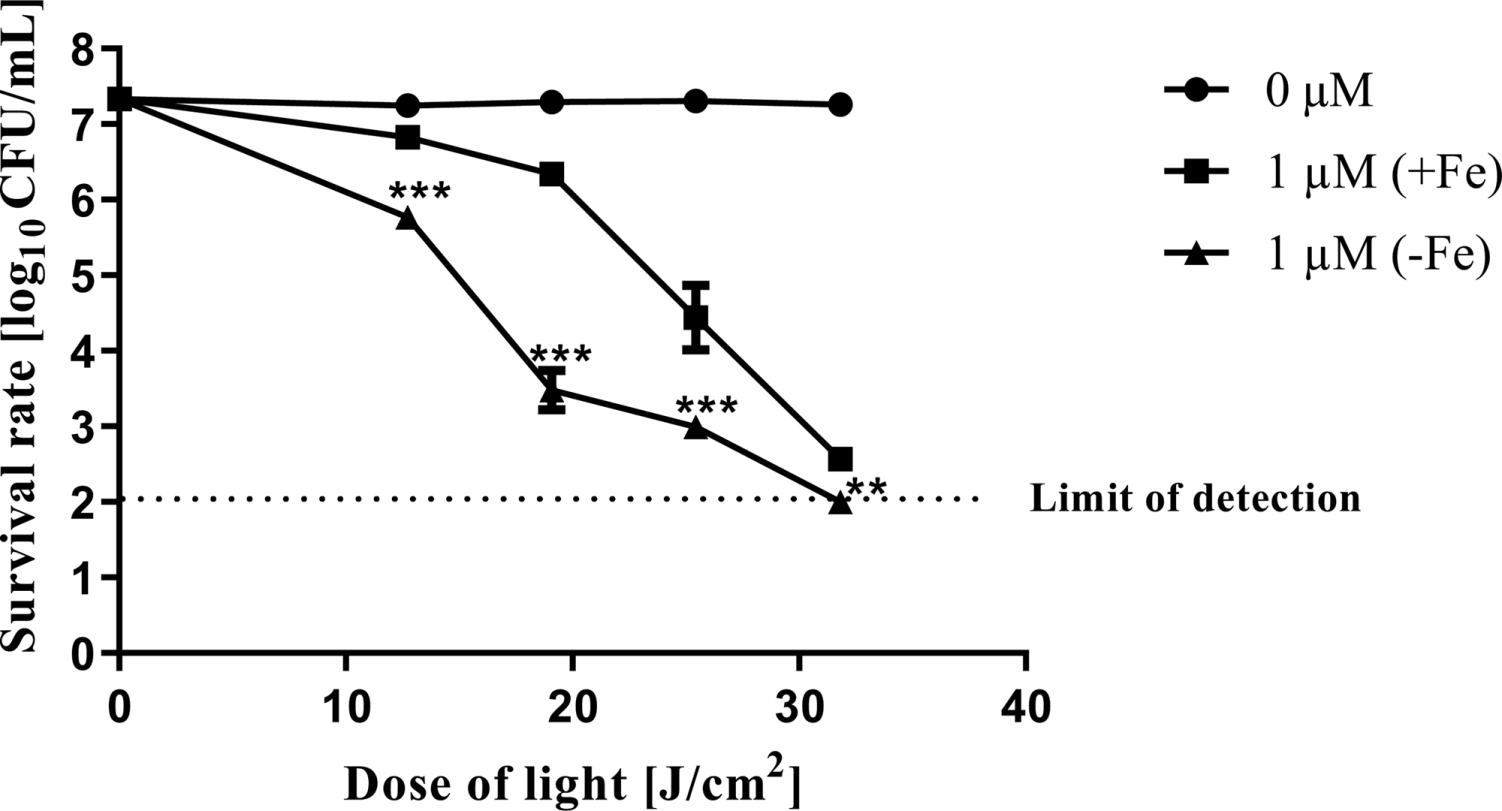
Photoinactivation of *S. aureus* 25923 with Ga^3+^MPIX in the presence and absence of iron. Overnight cultures of *S. aureus* 25923 strain were diluted to 0.5 MacF in medium with either the presence (+Fe) or absence (−Fe) of iron, then exposed to 0 or 1 μM Ga^3+^MPIX for 10 min at 37 °C, and then irradiated with different green LED light doses ranging from 0 to 31.8 J/cm^2^. Colony-forming units (CFU/mL) were estimated with serial dilutions of 10 μL aliquots of irradiated samples and plated on TSA agar. Plots present the reduction of log_10_ units of CFU/mL. The detection limit was 100 CFU/mL. Each experiment was performed in three biological experiments. The value is a mean of three separate experiments with bars as ± SD of the mean. Significance at the respective *p*-values is marked with asterisks [*p < 0.05; **p < 0.01; ***p < 0.001] with respect to 1 μM (+Fe) cells.

Limited availability of iron in the culture medium impacts the higher expression of certain iron-haem receptors.^37^ To check the hypothesis that the observed efficiency of Ga^3+^MPIX-mediated aPDI might be due to similar recognition of Ga^3+^MPIX molecules by haem receptors, the survival of the *S. aureus* 25923 reference strain was examined upon green LED light irradiation with Ga^3+^MPIX upon cultivation in the absence (−Fe) or presence (+Fe) of iron in the medium (Figure 5). In iron-rich medium, we observed a maximum reduction in the number of bacteria of 4.6 log_10_ units in CFU/mL for 1 μM at 31.8 J/cm^2^. Compared to these data, in an iron-depleted medium, the maximum viability of bacteria was noticeable at the limit of detection, which was a 5.3 log_10_ unit reduction in bacterial viability. Iron deficiency resulted in a higher efficiency of aPDI, with a 2.86 log_10_ difference in bacterial viability at a sublethal dose of 12.72 J/cm^2^ between two cultivation conditions. The efficiency of Ga^3+^MPIX-mediated aPDI is dependent on iron availability in the culture medium.

After phototreatment of *S. aureus* 25923 strain with Ga^3+^MPIX (1 μM, 25.4 J/cm^2^), surviving bacteria formed a small-colony variant (SCV) phenotype, which significantly differed from the original morphology. SCVs are classified as an atypical morphology with a lack of pigmentation, a smaller size, and a slower growth rate than the original cells. In iron-rich medium, only ∼10% of the total pool of surviving bacteria formed SCVs with the same pigmentation as original cells before treatment (Figure S4AB). However, under iron-poor conditions, SCV cells constituted nearly 60% of the total number of surviving bacteria. Moreover, constant, 20-h exposure to Ga^3+^MPIX during culturing (without light) had induced the SCV morphology in 100% of survived bacteria (Figure S4C). Continuous iron starvation and exposure to Ga^3+^MPIX promoted the SCV phenotype, which indicated the effect on iron metabolism. Interestingly, the long exposure to Ga^3+^MPIX cells was efficiently eradicated after green light irradiation (Figure S4D–F), indicating that SCVs are sensitive to Ga^3+^MPIX-mediated aPDI.

### Haem Has a Protective Effect on aPDI and the Accumulation of Ga^3+^MPIX

**Figure 6:**
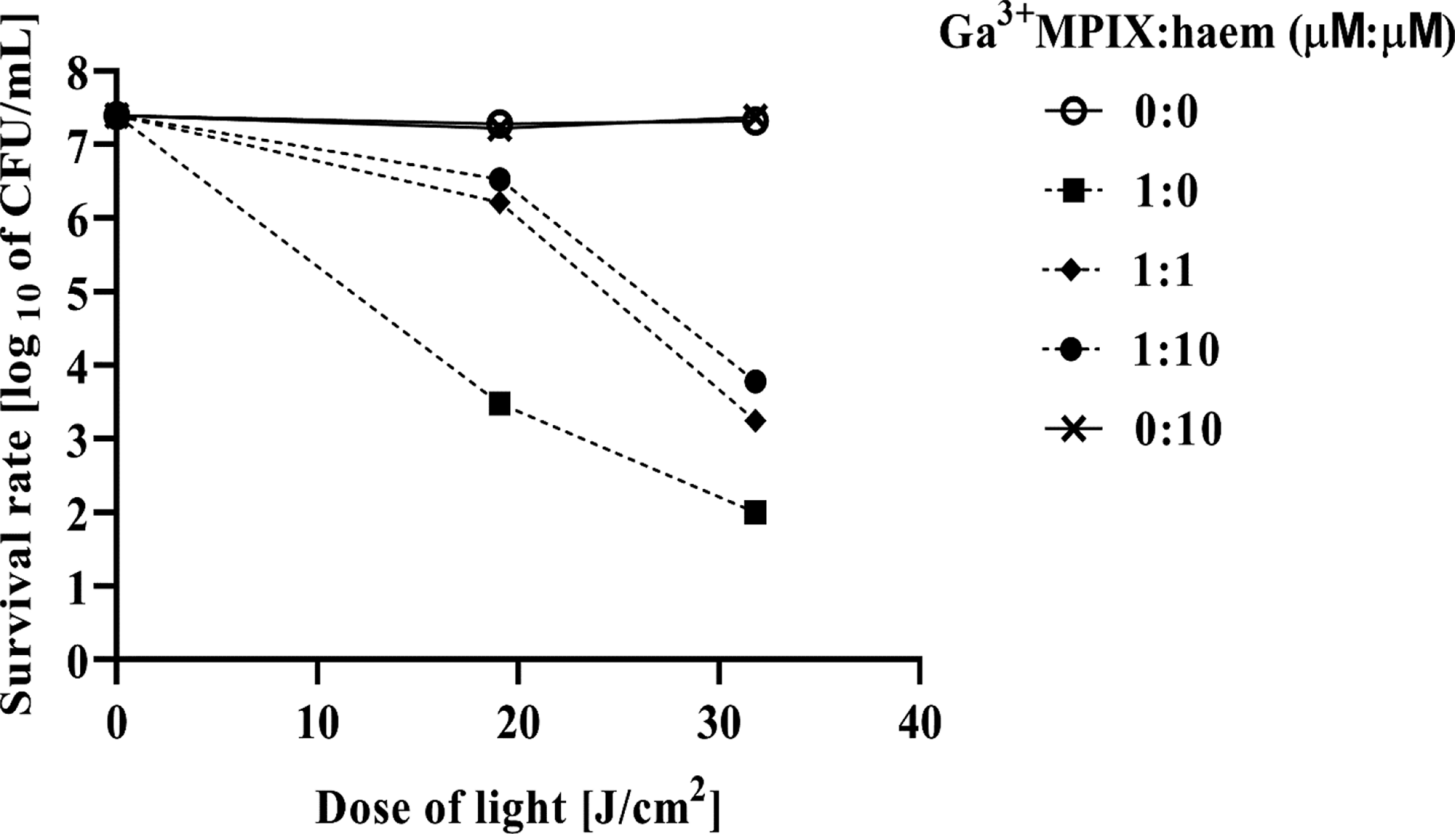
Protective effect of haem on Ga^3+^MPIX-mediated photodynamic treatment of the *S. aureus* ATCC 25923 strain. Iron-starved staphylococcal bacteria were incubated for 10 min with a mixture of Ga^3+^MPIX and haem at different ratios as indicated in the legend and then illuminated with a lower (19.08 J/cm^2^) or higher (31.8 J/cm^2^) dose of green light. Survival of bacteria was measured by serially diluting cells and counting the colony plated on agar plates after treatment (CFU/mL). The survival fraction is expressed as the number of CFU obtained after PDI treatment with respect to the number of CFU of nonlight-treated cells. The values are the means of three separate experiments. The value is a mean of three separate experiments with bars as ±SD of the mean.

Porphyrins with central metals in the oxidation state (III) might mimic structural haem and have an affinity to haem receptors.^24^ Ga^3+^MPIX might also be recognized by haem transporters and accumulate in a similar manner to haem. To determine whether the presence of haem influences the effectiveness of aPDI against *S. aureus*, we incubated bacterial cells with a mixture of haem and Ga^3+^MPIX and then irradiated them with lower (19.08 J/cm^2^) and higher (31.8 J/cm^2^) doses of light (Figure 6). By incubating cells with equal concentration or excess haem (1× or 10×), we observed a protective effect, that is, much fewer bacterial cells were photoinactivated compared to the situation when there was no haem in the reaction mixture, exhibiting a decrease of 1.25 log_10_ units in the reduction of CFU/mL for 1 and 1.7 log_10_ for a 10× higher haem concentration. This effect was especially observed with a lower light dose (decrease in CFU reduction of 2.73 log_10_ and 3 log_10_ for 1- or 10-fold haem concentration). We did not observe a difference between the 1-fold and 10-fold excess haem used. The observed protective effect of haem may be related to more efficient accumulation of haem in bacterial cells and competition of haem molecules with Ga^3+^MPIX for binding sites in/on cells.

**Figure 7:**
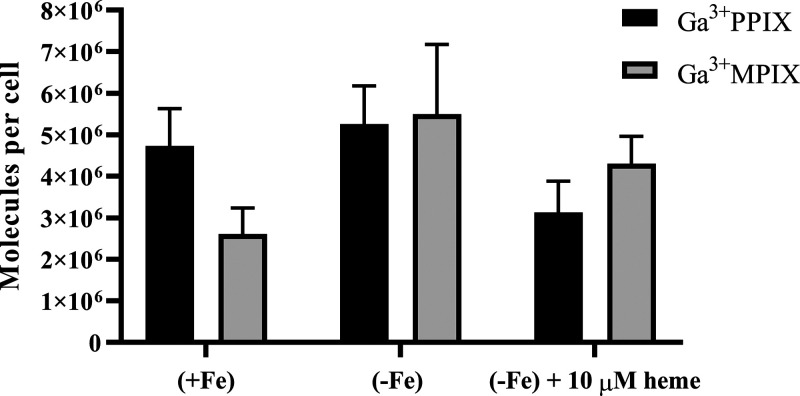
Uptake of Ga^3+^MPIX and Ga^3+^PPIX by the *S. aureus* ATCC 25923 strain in the presence (+Fe), absence (−Fe) of iron, or addition of haem. PS uptake was carried out in the presence of 10 μM of Ga^3+^MPIX or Ga^3+^PPIX. Bacterial cultures were incubated with the compound for 10 min at 37 °C with shaking followed by 24 h lysis as described in the Experimental Section, and then the fluorescence was measured. The value of the accumulated PS is represented as the number of molecules per cell, based on the standard curve of the compound in 0.1 M NaOH/1% SDS lysing buffer. The experiment was conducted in three independent biological repetitions.

Next, we examined whether Ga^3+^MPIX accumulation in *S. aureus* is dependent on iron availability in the culture medium (Figure 7). In the absence of iron in the medium (−Fe), the intracellular accumulation of Ga^3+^MPIX at 10 μM was 2.1 times higher than the accumulation at the same compound concentration in the presence of iron (+Fe). The accumulation of Ga^3+^MPIX was dose-dependent (data not shown). Iron starvation of *S. aureus* promotes higher accumulation of the compound. Based on our results, we checked whether the protective effect of haem in aPDI treatment would be reflected as lower intracellular accumulation of PS. *S. aureus* was incubated with a mixture of Ga^3+^MPIX and haem at a protective concentration of 10 μM in the absence of iron. The addition of haem resulted in a decrease in Ga^3+^MPIX uptake by 22% (1.2 × 10^6^ molecules per cell) with respect to PS accumulation alone in the absence of iron. The accumulation of Ga^3+^MPIX is also dependent on the presence of haem in the culture medium. The addition of the ligand for haem recognition receptors decreased the uptake of Ga^3+^MPIX by *S. aureus* cells, although statistical significance was not achieved in this situation. These results together with haem protection from Ga^3+^MPIX-mediated phototoxicity confirm that Ga^3+^MPIX is recognized in the same manner as haem.

Under the tested conditions, Ga^3+^PPIX also accumulated in *S. aureus* cells in an iron-dependent manner. In the absence of iron in the medium (−Fe), the intracellular accumulation of Ga^3+^PPIX at 10 μM was 1.11 times higher than the accumulation at the same compound concentration in the presence of iron (+Fe). The addition of haem reduced the Ga^3+^PPIX uptake by 40% (2.3 × 10^6^ molecules per cell) with respect to the accumulation of PS in the absence of iron. It is worth noting that under standard conditions, that is, in the presence of iron, bacterial cells accumulated more Ga^3+^PPIX compared to Ga^3+^MPIX, which may explain the greater toxicity of Ga^3+^PPIX in light-independent survival tests (Figure 3).

### Impairment in the HrtA Detoxification Efflux Pump Promotes Dark Toxicity of Ga^3+^MPIX

**Figure 8:**
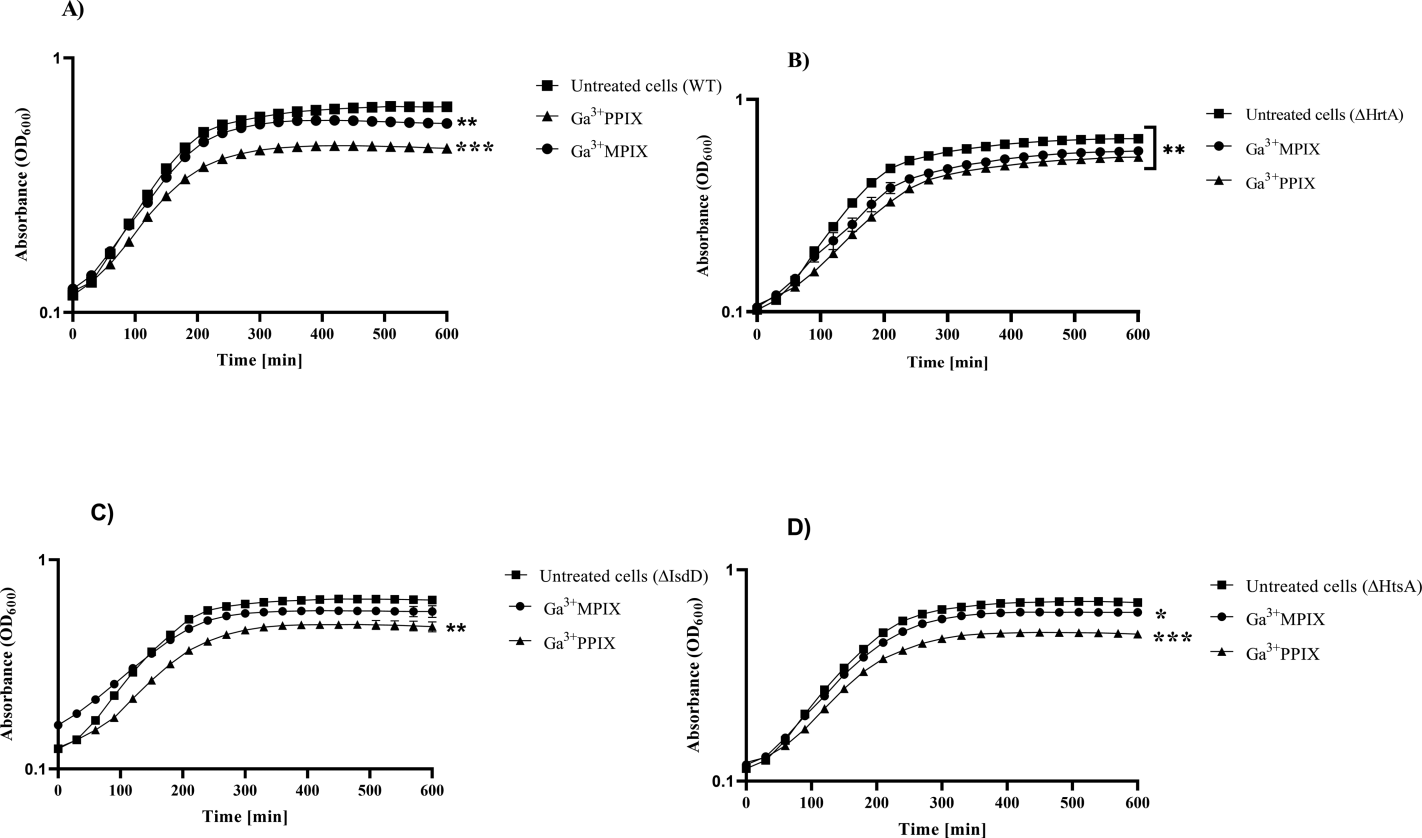
*S. aureus* Newman and its isogenic mutants (ΔHrtA, ΔIsdD, and ΔHtsA) grown under exposure to MP and non-MP compounds in the presence of iron in medium. Overnight cultures of *S. aureus* Newman WT (A), ΔHrtA (B), ΔIsdD (C), and ΔHtsA (D) in the iron-rich medium were diluted 1:20 and exposed for 10 μM of Ga^3+^MPIX, Ga^3+^PPIX, or left untreated (untreated cells). Growth of each condition was monitored by measurement of optical density at 600 nm (OD_600_) on an Envision plate reader. Experiment was conducted on three independent biological repetitions. Error bars represent the SD values. Significance at the respective *p*-values is marked with asterisks [*p < 0.05; **p < 0.01; ***p < 0.001] with respect to untreated cells of each phenotype.

As the presence of haem influenced the level of Ga^3+^MPIX accumulation and aPDI efficiency, we hypothesized that haem acquisition machinery might also be involved in PS recognition. To understand the molecular mechanism responsible for the uptake and detoxification of gallium conjugates, we analyzed the growth of *S. aureus* Newman (WT) and its isogenic mutants deprived of genes engaged in haem uptake (ΔIsdD and ΔHtsA) and detoxification (ΔHrtA). The growth curves of *S. aureus* of each phenotype were analyzed after constant exposure to gallium MPs such as Ga^3+^MPIX or Ga^3+^PPIX (Figure 8) in an iron-rich environment. We compared several growth parameters such as maximum specific growth rate (μ_max_), duplication time (*T*_d_) of the exponential phase and for the stationary phase: time to reach the stationary phase, and maximum density (*A*_max_) in each mutant after treatment with gallium compounds (Supplementary Table S4). Both compounds Ga^3+^PPIX and Ga^3+^MPIX reduced the μ_max_ of each strain studied in a similar manner, that is, the highest inhibition was observed for ΔHrtA and ΔIsdD. Each treatment was compared to the control—untreated cells of each mutant (calculated as 100%). Despite gene deletion, untreated mutants achieved a similar growth rate to untreated WT. Under exposure to Ga^3+^PPIX, the growth of each strain at the end of the exponential phase (after 270 min of analysis, taken as the point of inhibition of the exponential growth—cutoff point) was estimated to be 72–77% of the growth of respective untreated controls, which indicated the higher toxicity of this compound. However, the main difference between mutants’ growth was observed under Ga^3+^MPIX exposure. The WT, ΔIsdD, and ΔHtsA strain grew up to 90–93% in a medium containing Ga^3+^MPIX, whereas in ΔHrtA it was only 82%, thus indicating that Ga^3+^MPIX was the most toxic for this mutant. Impairment in the HrtAB efflux pump resulted in the more pronounced toxicity of Ga^3+^MPIX toward *S. aureus* in comparison to other phenotypes. Interestingly, a clear effect in the form of a significant extension of the doubling time (*T*_d_) was also observed in relation to ΔIsdD, where Ga^3+^MPIX toxicity resulted in an increased *T*_d_ parameter from 1.46 to 2.06 OD_600_/h.

### HrtA-Lacking Mutant Is the Most Sensitive Phenotype to Ga^3+^MPIX-Mediated aPDI

**Figure 9:**
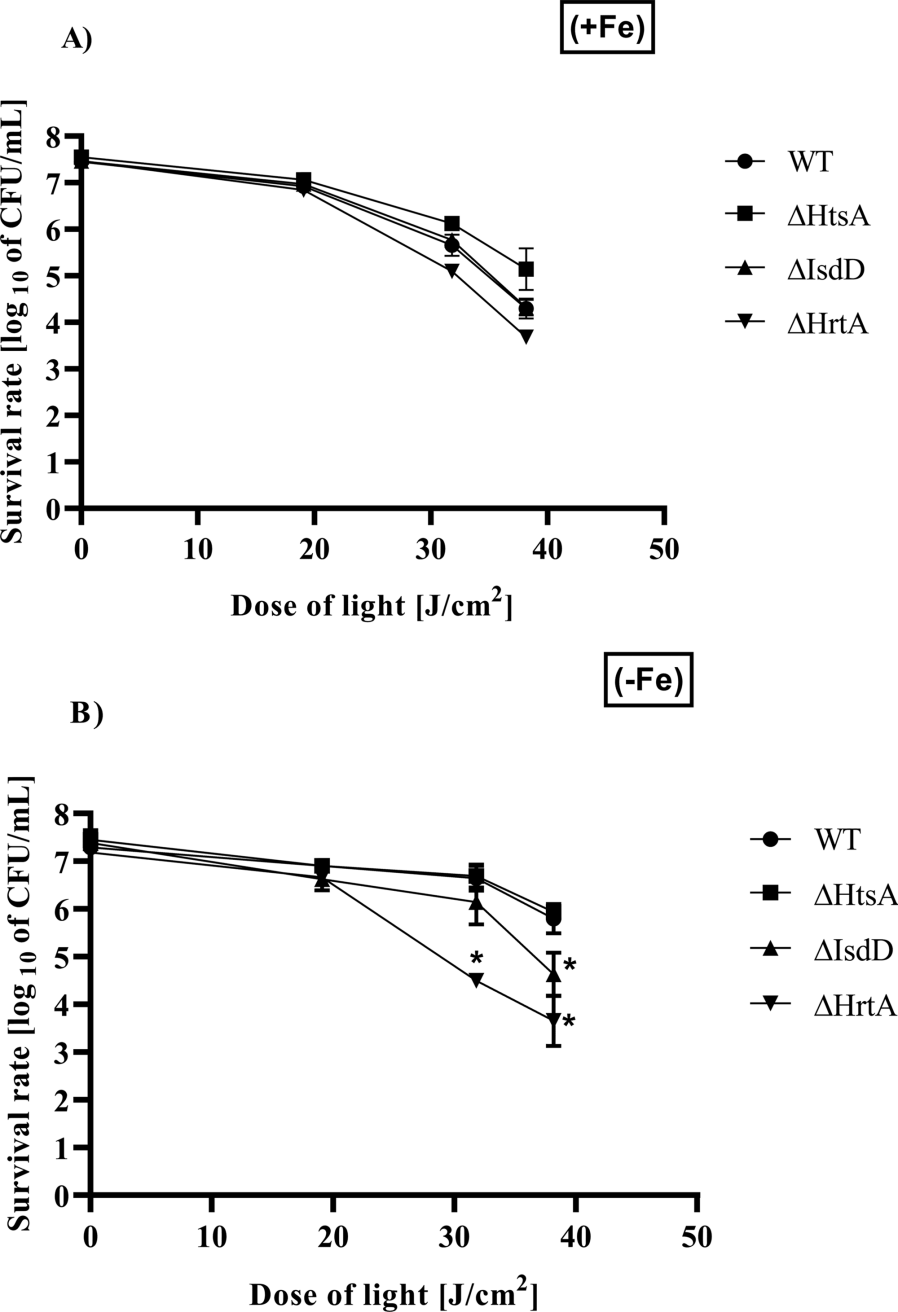
Phototreatment of *S. aureus* Newman and its isogenic mutants (ΔHrtA, ΔIsdD, and ΔHtsA) with 10 μM Ga^3+^MPIX under green light irradiation in the presence (A) and absence (B) of Fe^3+^ in the medium. After incubation with 10 μM Ga^3+^MPIX, the analyzed strains were subjected to green light (19.8–38.16 J/cm^2^). Bacterial survivals were measured by serially diluting cells and counting the CFUs plated on agar plates after treatment. Each experiment was conducted in three independent biological experiments for every condition. The values represent the means of survived bacteria with bars as ±SD of the mean. Significance at the respective *p*-values is marked with asterisks [*p < 0.001] with respect to WT cells.

In our previous studies on aPDI on the Newman WT strain and its isogenic mutants, ΔHrtA was the most susceptible to PPIX-mediated aPDI.^48^ Here, we were interested in whether differences among haem transport mutants could also be observed in the sensitivity to Ga^3+^MPIX-based aPDI. Therefore, we performed aPDI against *S. aureus* Newman and its isogenic mutants (10 μM Ga^3+^MPIX, 19.8–38.16 J/cm^2^) in the presence (Figure 9A) and absence of iron (Figure 9B). We increased the dose of green light to 38.16 J/cm^2^ to observe more pronounced differences between phenotypes. In the presence of iron, the maximal bacterial reduction in CFU/mL was observed as follows: 3.78 – ΔHrtA, 3.15 – ΔIsdD, 2.5 – ΔhtsA, and 3.15 log_10_ units for WT. In the absence of iron, the maximal reduction in CFU/mL was estimated to be 3.5 – ΔHrtA, 2.75 – ΔIsdD, 1.44 – ΔHtsA, and 1.5 log_10_ units for WT. Interestingly, the absence of Fe^3+^ in the medium did not significantly increase the efficiency of aPDI. Under both cultivation conditions, the ΔHrtA mutant presented the most PDI-sensitive phenotype. Moreover, the ΔHtsA mutant was the most resistant to aPDI treatment among all phenotypes. Taking these results together, impairment in HrtA ATPase in the HrtAB detoxification system provides higher sensitivity to Ga^3+^MPIX-based aPDI.

**Figure 10:**
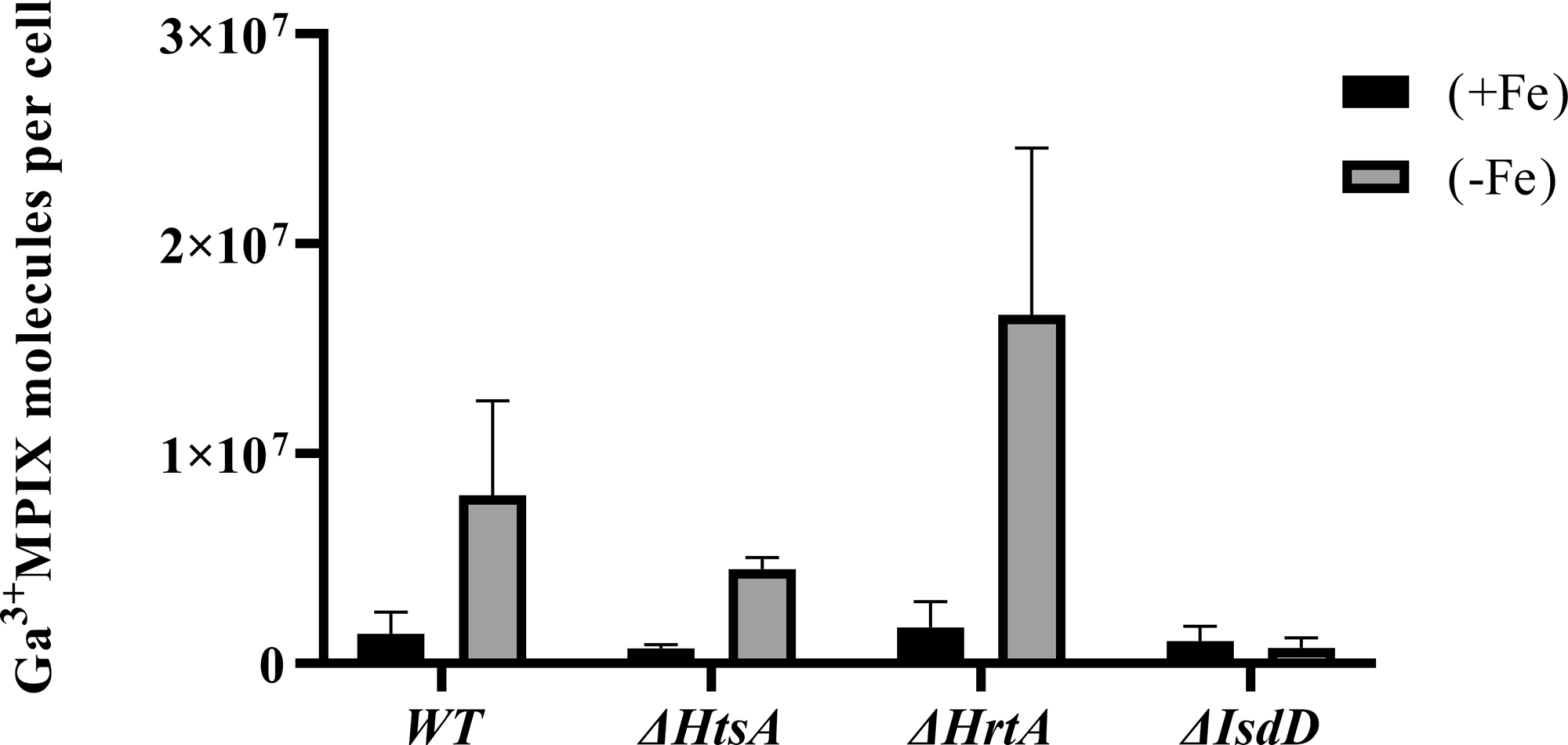
Ga^3+^MPIX uptake in *S. aureus* Newman and its isogenic mutants (ΔHrtA, ΔIsdD, and ΔHtsA) in the presence (+Fe) or absence (−Fe) of Fe ^3+^ in the medium. Overnight bacterial cultures of each phenotype were diluted and incubated with a PS for 2 h at 37 °C with shaking and then, washed twice with PBS buffer and suspended in 0.1 M NaOH/1% SDS solution. After 24 h of incubation, the fluorescence of the lysate was measured (ex/em 398/573 nm). The values represented in the graph are the mean with SD of triple biological repetition for every strain in each condition.

To understand the mechanism of the superior efficiency of aPDI in the ΔHrtA mutant, the accumulation of Ga^3+^MPIX was investigated in each phenotype. Briefly, bacterial cells were cultivated in different iron contents at the stationary phase of growth, diluted, and then incubated in the dark with PS for 2 h at 37 °C with shaking. Then, bacterial lysates were prepared and measured as described in the Experimental Section. In the presence of iron (+Fe), we did not observe significant differences in accumulation between the studied phenotypes (Figure 10). Iron starvation (−Fe) increased PS uptake in comparison to iron presence in the media for each phenotype, except for ΔIsdD, in which the accumulation remained at the same level. Ga^3+^MPIX accumulation in ΔHrtA was 2-fold higher than that in the WT strain in the absence of iron. Additionally, the uptake of the PS was decreased by approximately 50% for ΔHtsA and 90% by ΔIsdD compared to the WT strain.

The use of fluorescence microscopy did not give unequivocal results; that is, a stronger fluorescence signal was observed for ΔHrtA and WT under both (+Fe) and (−Fe) conditions (Figures S5 and S6). In contrast, ΔIsdD and ΔHtsA showed a stronger fluorescence signal under (−Fe) compared to (+Fe) (Figure S7). This indicates that the presence of Fe^3+^ influences Ga^3+^MPIX uptake rather than removal from the cell.

### Ga^3+^MPIX Does Not Promote Extensive and Prolonged Cytotoxicity or Phototoxicity against Human Keratinocytes

**Figure 11:**
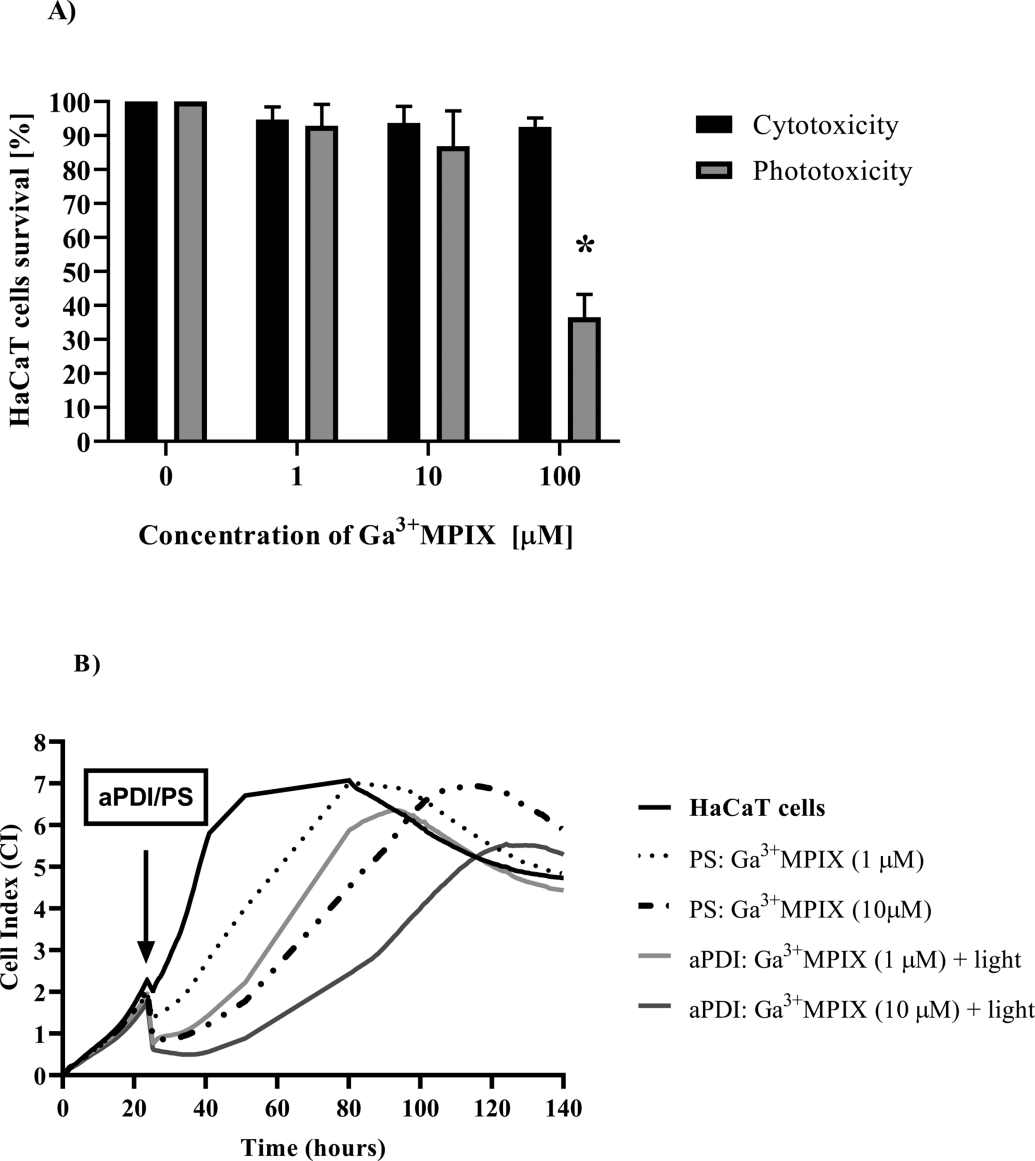
Effect of aPDI with Ga^3+^MPIX on the HaCaT cell line model. (A) MTT cell viability assay. HaCaT cells were exposed to various concentrations of Ga^3+^MPIX. Control cells (0 μM) to which no test compound was added. After incubation with Ga^3+^MPIX, cells were either irradiated with green light (31.8 J/cm^2^) represented by gray bars (Phototoxicity) or kept simultaneously in the dark (black bars for cytotoxicity). Each result is the mean ± SD of the mean. Significance at the respective *p*-values is marked with asterisks (∗ *p* < 0.05) for untreated cells in each condition. (B) Cell growth dynamics. Cells were seeded at 10^4^ cells/well and after obtaining a CI of 2, cells were treated with Ga^3+^MPIX in the dark and incubated at 37 °C for 10 min. The samples were then illuminated with a green light dose of 31.8 J/cm^2^, while the HaCaT cells or PS only treatment was allowed to incubate in the dark at room temperature. The CI (represented as the Y axis) was measured for each condition every 10 min. The *x*-axis shows the experimental duration in hours. The values presented are the average of the 7 technical repetitions.

PS safety toward eukaryotic cells is a crucial factor for optimization and further applications of photoinactivation protocols. We examined the phototoxicity and cytotoxicity of Ga^3+^MPIX against human keratinocytes. Variations in concentrations of Ga^3+^MPIX were used for treatment under both light and dark conditions with twice wash step (Figure 11) and once wash step (Figure S8). The highest dose of green light (31.8 J/cm^2^) was selected, which corresponds to the bactericidal effect toward several *S. aureus* strains. Additionally, we increased the concentration of the PS up to 100 μM to ensure its high excess. Based on the MTT assay results in Figure 11A, the viability of cells was affected by neither the presence of Ga^3+^MPIX alone (94.73 and 93.7% survival upon 1 and 10 μM) nor under green light irradiation (92.87 and 86.9% survival upon 1 or 10 μM). Cell survival estimated at ∼80% is considered acceptable, modest toxicity to eukaryotic cells.^17^ Increasing the compound concentration to 100 μM under green light showed significantly increased phototoxicity toward HaCaT cells (36.57% cell survival) in comparison to cells exposed only to light without the PS. In the dark, 100 μM Ga^3+^MPIX had no significant impact on cell viability (estimated 92.55% survival). Ga^3+^MPIX exhibited relative safety on HaCaT cell survival at 31.8 J/cm^2^ green light irradiation up to 10 μM concentration.

However, cytotoxicity and phototoxicity were more pronounced when the cells were washed once compared to two times (Supplementary Figure S8A). After washing the cells once, a concentration of 100 μM Ga^3+^MPIX (31.8 J/cm^2^) almost completely reduced the number of the viable HaCaT cells, while approximately 50% of the cells survived the treatment with 10 μM Ga^3+^MPIX (31.8 J/cm^2^). The light-independent cytotoxicity decreased to approx 80% (100 μM) compared to the twice-washing procedure when this value was negligible.

However, the MTT assay has some methodological limitations, such as measuring only at a specific time point. Additionally, the cell proliferation rate and morphology were not taken into consideration. RTCA on E-plates is a method consisting of electrographic detection of the cell number, morphology, adhesion, and rate of proliferation under experimental conditions. Based on real-time cell growth dynamic curves (Figure 11B), we observed a slower proliferation rate of HaCaT cells after treatment with 1 or 10 μM Ga^3+^MPIX under either dark or illumination conditions. Untreated cells resumed growth and reached the plateau phase at approximately the 60th h. Ga^3+^MPPX dark-treated cells reached the plateau phase at approximately 85 h at 1 μM and 100 h at 10 μM. After photodynamic treatment (1 μM Ga^3+^MPIX, 31.8 J/cm^2^), HaCaT cells reached a plateau phase at the same hour as dark-treated cells at the same concentration of Ga^3+^MPIX. A higher difference between light-exposed and dark-kept cells was observed in 10 μM Ga^3+^MPIX, where the cell recovery phase was reduced, and the plateau phase was detected after 120 h. The cell proliferation rate and recovery were lowered in a concentration-dependent manner. The illuminated PS provided higher inhibition of HaCaT growth dynamics than the PS in the dark. During aPDI treatment, a fraction of the cells was damaged, but in most aPDI-treated cells, the damage was repaired, and the cells continued to grow and divide. Growth dynamics of HaCaT cells after aPDI with one wash step instead of the two washes (Figure S8B) was similar, showing the highest phototoxicity for 10 μM Ga^3+^MPIX. Thus, we claim that Ga^3+^MPIX alone or under photodynamic treatment does not promote extensive and prolonged cytotoxicity or phototoxicity against human keratinocytes.

## Discussion

Targeted PS recognition in aPDI is a novel trend of action against microorganisms, which developed from the origin of cancer cell phototreatments.^51^ Based on the Trojan Horse strategy of action, gallium MPs could be potent antimicrobial agents. Recognized by bacterial cells in the same manner as the natural ligand haem, gallium compounds might interrupt haem/iron metabolism.^23^ Many studies have only confirmed the activity of gallium MPs toward several ESKAPE pathogens, including antimicrobial and antibiofilm action.^25,26,28,29^ Ga^3+^MPIX resulted in the same MIC value for *S. aureus* (1.6 μg/mL) as Ga^3+^PPIX.^23^ Interestingly, porphyrins without metal ions (i.e., PPIX and MPPIX) were not efficient inhibitors of bacterial growth.^23^ The rationale for choosing Ga^3+^MPIX for our research stems from studies on metalloporphyrins which showed effective induction of HrtAB, a molecular haem transport system. Previous studies on metalloporphyrin toxicity and the molecular mechanism underlying this process have shown that the HssRS—haem detoxification system is quite widely activated by metalloporphyrins, while the HrtAB efflux pump will only be sensitive to certain metalloporphyrins, in particular Ga^3+^PPIX or Mn^3+^PPIX.^52^ These observations prompted us to investigate the accumulation in cells and efficacy of the Ga^3+^MPIX compound in aPDI, excitation in a spectrum which is not commonly used in research, that is, green light. In particular, we were interested in studying the functionality of the vinyl group (protoporphyrin IX) to ethyl group (mesoporphyrin IX) change in the porphyrin macrocycle, as from the literature to date, the modification of the side compound chains was presented with minor interest, such as changes inside the porphyrin ring (i.e., central metal^34,53^). We hypothesized that despite this difference, Ga^3+^MPIX might be recognized by haem receptors, and consequently, released gallium ions induce dark toxicity similar to Ga^3+^PPIX. The dependence of the accumulation of Ga^3+^MPIX in bacterial cells on iron and the protective role of haem in aPDI indicates competition for binding with haem receptors and shows that specific haem transport systems into or out of the cell may play a role in the photoinactivation process. This is because bacteria do not distinguish some MPs from their natural ligand, the haem, and use them in their natural metabolic processes leading to inhibition of cell growth and death. Previously published data showed that only some MPs can do this, for example, Ga^3+^PPIX^34^ and Mn^3+^PPIX.^52^ Here, we showed that Ga^3+^MPIX can also behave in a similar manner. Furthermore, the molecular structure of the MPs to be used as a substrate for the targeted delivery to bacterial cells is of primary importance. Previously published data indicated that porphyrin ring ion groups (carboxyl) have been shown to be important for interactions with haem uptake systems. Replacing them with esters that cannot be ionized has resulted in the loss of selective uptake by haem extraction systems to the advantage of nonspecific uptake.^34^ In our experiments, the transition from more hydrophobic vinyl (in Ga^3+^PPIX) to less hydrophobic ethyl (in Ga^3+^MPIX) groups in the porphyrin macrocycle resulted in several different behaviors, including solubility, absorption, but also accumulation and photoinactivation.

The two molecules Ga^3+^MPIX and Ga^3+^PPIX are similar in terms of their general structure (the only difference being vinyl vs ethyl groups) and production of singlet oxygen (in aqueous solution), yet they differ in light-dependent (significantly) and light-independent (slightly) activity. The observed difference between the activity of both compounds without light is most likely due to the more effective accumulation of Ga^3+^PPIX than Ga^3+^MPIX (Figure 7) which results in a greater reduction in the growth rate of bacterial cells (Figures 3 and 8). The chemical modification of the porphyrin macrocycle seems to alter the potency of these compounds to regulate haem metabolism in vivo. Those molecules may differently react with their molecular targets in the bacterial cells, which results in the observed differences in the light-independent process. However, the issue of light-dependent action of the two compounds is more related to the biophysical properties of the compounds themselves. First, the differences in absorption spectra, although slight, are nevertheless noticeable. For Ga^3+^PPIX, the absorption maxima in the Q band region are λ_max_ = 541 nm and λ_max_ = 580 nm, while the analogous Ga^3+^MPIX maxima are λ_max_ = 532 and λ_max_ = 570 nm and are shifted toward shorter wavelengths. As a result, they better match the emission spectrum of the LED lights we used. The second and more important element explaining the different effectiveness of both compounds is the solubility in aqueous solutions. As is well known, porphyrin compounds do not readily dissolve in aqueous solvents. In our experiments, Ga^3+^MPIX dissolved much better in the aqueous solution (0.1 M NaOH titrated to PBS) than Ga^3+^PPIX. Ga^3+^PPIX in 0.1 M NaOH titrated to PBS generated a double peak, which is most likely responsible for the appearance of oligomeric forms in the solution (Figure S1). The addition of 50% DMSO shifted the equilibrium of monomeric and oligomeric forms toward the one resulting in a higher absorption signal (Figure S1) and most likely corresponding to a monomer.^54^ Thus, the difference in the structure of both compounds (ethyl vs. vinyl groups) has a significant impact on their solubility in an aqueous solution. This feature is extremely important from a clinical point of view. From the available literature data, it appears that the difference between the activities of porphyrin and mesoporphyrin was not that significant (estimated as at most 1 log_10_)^53,55^ as observed in our experimental setup. It is worth noticing, however, that the elsewhere tested compounds were dissolved in solutions with the addition of DMSO, which strongly affects the solubility of protoporphyrin derivatives. Because of the potential clinical use of aPDI, photosensitizing compounds should be dissolved in aqueous solutions, avoiding the use of organic solvents. In this case, Ga^3+^MPIX meets this requirement and Ga^3+^PPIX does not.

The recent study of Morales-de-Echegaray et al. revealed the dual functionality of Ga^3+^PPIX. Despite gallium toxicity, these compounds might also act as PSs in aPDI upon blue light irradiation (405 nm, 140 mW/cm^2^) with maximal staphylococcal reduction >6 log_10_ of bacterial viability.^34^ The photodestruction was characterized as rapid (after 10 s of irradiation), and authors suggested that high-affinity surface hemin receptors such as the Isd system might have a role in the process.^34^ Moreover, the Skaar group recently showed that anti-Isd monoclonal antibody together with aPDI proved to be effective against drug-resistant *S. aureus* in a murine model of soft tissue infections.^56^ Based on the literature, the lack of iron upregulates the gene expression of haem receptors of the Isd system on the bacterial surface.^23^ This might be the possible explanation for the higher aPDI efficiency, where Ga^3+^MPIX is recognized by Isd or Hts similarly to haem. We confirmed this by studying aPDI in an Fe-dependent manner (Figure 5), and we observed the protective effect of haem in the process of aPDI (Figure 6) or accumulation of Ga^3+^MPIX (Figure 7). In our study, the impairment of haem surface receptors, such as IsdD or HtsA, was manifested by a reduction in Ga^3+^MPIX accumulation as measured by two fluorescence methods. Based on these results, we hypothesized that despite the ethyl instead of vinyl groups in the side chains of the porphyrin structure, Ga^3+^MPIX is recognized by haem uptake receptors (mainly Isd) and is a competitor of haem. Impairment in the HrtA component of the efflux pump potentiated the effect of aPDI, and this effect was the most visible of all mutants tested, although many factors could influence its efficacy.^57^ We previously reported that increased aPDI efficacy in ΔHrtA mutant can also be observed because of physical changes in the membrane composition and not the lack of functional protein.^48^ The lipid content of the bacterial membrane might also contribute to the observed result in Ga^3+^MPIX-mediated aPDI.^57^ However, the substrate of the HrtAB efflux pump or the molecular mechanism of detoxification of gallium MPs is currently unknown, and further studies in this area should be encouraged.

Most studies on the antimicrobial activity of Ga^3+^PPIX were conducted in a light-independent manner.^23,27−29^ Light-dependent action was demonstrated only for blue light with excitation in the Soret band (∼405 nm).^34,35^ In this study, we propose the excitation of Ga^3+^MPIX within one of the Q-bands using green light. Green light ensures deeper tissue penetration than blue light while preserving sufficient energy to activate the compound. Moreover, the green LED lamp (λ_max_ = 522 nm) exhibits a low light toxicity level toward bacterial cells themselves, as demonstrated in our current study. In light-only treatments, there was no pronounced excitation of endogenous porphyrins, so the aPDI effect was related to only exogenously applied PSs. In the case of Ga^3+^MPIX-mediated aPDI, we observed a maximal reduction in bacterial viability in the range of 3–6 log_10_ (2–5 log_10_ after a wash). This indicates that Ga^3+^MPIX has good efficiency against *S. aureus* compared to other Ga^3+^PPIX excited with a shallow penetrating blue light.^53,55^ Because of the use of green light, it is potentially possible to photoinactivate bacteria that penetrate deeper layers of the skin than blue light. Verifying such an approach, however, would require additional research on more complex in vivo models.

Iron starvation alters bacterial metabolism by changes in the expression of several staphylococcal genes involved in iron acquisition, glycolysis, and virulence via a Fur-mediated mechanism. These changes are related to different colony phenotypes known as SCVs.^58^ Based on previous research, MPs such as Ga^3+^PPIX induced this phenotype by inhibiting respiration or inducing oxidative stress, which was indistinguishable from genetic SCVs.^52^ The SCV phenotype appears to be responsible for chronic and recurrent infections and is also highly resistant to antibiotics.^59^ We observed the presence of the SCV phenotype during 16–20 h of light-independent, constant cultivation of bacteria with Ga^3+^MPIX (Figure S4A–C). At the same time, it is worth noting that the exposure to Ga^3+^MPIX caused sensitization of SCVs to light and, as a result, the eradication of microbial cells upon green light (Figure S4D–F).

Red light is usually employed in photodynamic applications of porphyrins because of the depth of tissue penetration (dermis layers). The use of green light to treat superficial skin lesions seems particularly attractive. Because green light does not penetrate as deeply into the skin as red light causes much less pain during the irradiation in patients.^60^ It penetrates only the epidermis without irritating the nerve fibers. Ga^3+^MPIX can be efficiently activated by green light without causing extensive and prolonged phototoxicity against HaCaT cells. Although, under our experimental conditions the observed phototoxicity seems to be higher compared to Ga^3+^PPIX published by others, where only minor phototoxicity was observed after blue light activation.^34^ Ga^3+^MPIX under photodynamic treatment does not promote extensive phototoxicity against human keratinocytes; however, cells exhibit a slower proliferation rate than untreated cells. The cells with moderate or none photodamage resume growth and divide thus indicating that there is a place here for “therapeutic window.” The observed growth delay was not prolonged. These in vitro experiments confirmed the safety of Ga^3+^MPIX-mediated aPDI application to further studies on ex vivo models (e.g., porcine skin) or in vivo models (e.g., mouse models).

Research on photosensitizing compounds using natural bacterial cell transport systems is an extremely interesting path in the development of targeted PDI. The Trojan Horse strategy based on haem analogues, proposed years ago,^23^ shows that discrete changes in the structure of PS molecules can significantly affect its properties and enable further development of this strategy against *S. aureus* infections.

## Conclusions

In conclusion, Ga^3+^MPIX acts in two ways: independent of light (by blocking iron metabolism) or dependent on light (photodynamic action). This type of two-way mechanism of action provides very good protection against the selection of *S. aureus* mutants resistant to photodestruction. This study demonstrated that green light excitation of Ga^3+^MPIX in the Q band absorption area resulted in eradication of bacteria (reduction >5log_10_ CFU/mL) while maintaining relative safety for the eukaryotic cells tested. We have demonstrated that Ga^3+^MPIX-mediated aPDI exhibits Fe-dependent efficiency, and haem has a protective effect, indicating the importance of specific haem transport systems in the aPDI system under study. We have shown that Ga^3+^MPIX, with ethyl groups in the porphyrin macrocycle instead of vinyl groups present in Ga^3+^PPIX, can be recognized by haem uptake machinery, preferably by Isd. Impairment in the HrtA efflux pump turned out to be the most sensitive to aPDI with Ga^3+^MPIX. This study showed that despite the structural changes around the porphyrin ring, Ga^3+^MPIX was able to sustain its dual functionality. In addition, these changes can improve other properties of the compound, such as a higher efficiency in the photodynamic action.

## Supporting Information Available

Antibiotic resistance profiles and parameters describing the growth rate of the tested *S. aureus* strains, information on Ga^3+^MPIX and Ga^3+^PPIX absorbance spectra, ROS detection results with fluorescent probes in vitro and in vivo using ROS quenchers, photoinactivation of SCVs, accumulation of Ga^3+^MPIX under a confocal microscope, and cyto- and phototoxicity tests against *S. aureus* and HaCaT cells (single wash condition) (PDF)
