# Supplementary material for: Gallium Mesoporphyrin IX-Mediated Photodestruction: A Pharmacological Trojan Horse Strategy To Eliminate Multidrug-Resistant Staphylococcus aureus
Source: Mol Pharm. 2022 Apr 13;19(5):1434–48. doi: 10.1021/acs.molpharmaceut.1c00993 (PMC9066410; doi:10.1021/acs.molpharmaceut.1c00993)
Supplement: Supplementary file 1 — mp1c00993_si_001.pdf [file mp1c00993_si_001.pdf]

Supplementary Materials to Gallium mesoporphyrin IX mediated photodestruction: a pharmacological Trojan horse strategy to eliminate multidrug-resistant *Staphylococcus aureus*

Klaudia Michalska K, Michał Rychłowski[2], Martyna Krupińska[1], Grzegorz Szewczyk[3], Tadeusz Sarna[3], Joanna Nakonieczna[1]

Bacterial strains characterization 2

Chemicals used in the study...3

Gallium MPs delayed staphylococcal growth light-independently...4

ROS detection and Quenchers in Ga^{3+}MPIX phototreatment 5

Phototreatment of *S. aureus* with Ga^{3+}MPIX or Ga^{3+}PPIX reduced bacterial viability after additional wash-step 6

Ga^{3+}MPIX-mediated photoinactivation of SCVs 7

Impairment in the HrtA detoxification efflux pump promotes dark toxicity of Ga^{3+}MPIX..........8

Ga^{3+}MPIX accumulation under confocal microscopic image...9

$Ga3^{+}$MPIX does not promote extensive and prolonged cytotoxicity or phototoxicity against human keratinocytes...12

Bacterial strains characterization

Table S1. Drug resistance of *S. aureus* 1814/06 and 4046/13 MDR strains used in this study.

Antibiotic	*S. aureus* 1814/06	*S. aureus* 4046/13
FOX	**R**	**R**
ERY	**R**	**R**
CLI	**R**	**S**
QDA	**S**	**S**
MUP	**S**	**S**
SXT	**S**	**R**
VAN	**S**	**S**
TEI	**S**	**S**
TET	**R**	**R**
DK	**R**	**R**
TGC	**S**	**S**
MIN	**R**	**R**
CIP	**S**	**R**
GEN	**R**	**R**
FA	**S**	**S**
RIF	**S**	**S**
TLV	**S**	**S**
DAP	**S**	**S**

Legend: R- Resistance S- Susceptible; FOX- fosfomycyn, ERY-erytromycyn, CLI- clindamycin , QDA- quinupristin-dalfopristin, MUP-mupirocin , SXT- trimethoprim-sulfamethoxazole, VAN- vancomycin, TEI- teicoplanin; TET- tetracycline; DK-Dicloxacillin ;TGC- tigecycline; ***MIN****- minocycline; CIP- ciprofloxacin; GEN- gentamycin; FA- fusidic acid; RIF-rifampicin; TLV- telavancin , DAP- daptomycin*

Chemicals used in the study

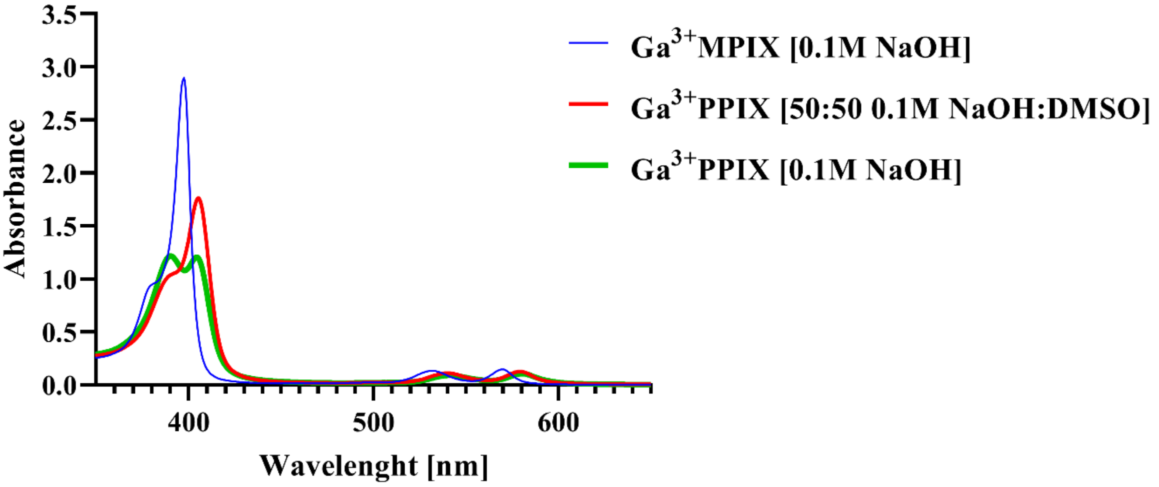

Figure S1. Absorbance spectra of Ga^{3+}MPIX or Ga^{3+}PPIX in different solvents. Absorbance spectra of 10 µM stock of Ga^{3+}MPIX or Ga^{3+}PPIX initially diluted in different type of solvents (0.1 M NaOH or 0.1 M NaOH:DMSO) were dissolved in PBS buffer. Stock solution (1 mM) Ga^{3+}MPIX in 0.1 M NaOH (blue line) was dissolved in PBS to a working concentration of 10 µM. stock solution (1mM) Ga^{3+}PPIX in an organic solution 50:50 (v: v) 0.1M NaOH: DMSO (red line) or in an aqueous solvent of 0.1M NaOH (green line) were dissolved in PBS to a working solution of 10 µM. Each spectrum recorded is the mean of three independent replicates.

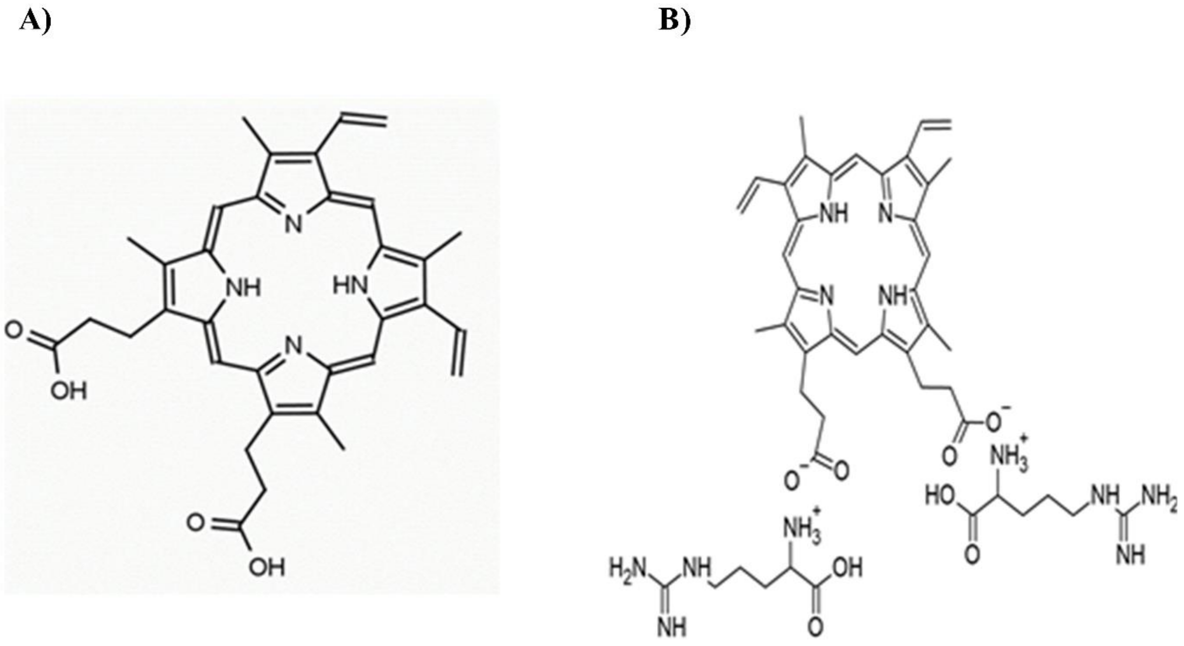

PPIX
Formula: $C_{34}H_{34}N_4O_4$
Mass: 562.658 g/mol

PPIXArg₂
Formula: $C_{46}H_{62}N_{14}O_8$
Mass: 911.061 g/mol

Figure S2. Molecular characteristic of compounds used in this study. A) Protoporphyrin IX (PPIX) **B)** Protoporphyrin IX diarginate ($PPArg_2$).

Gallium MPs delayed staphylococcal growth light-independently

Table S2. Staphylococcal growth under exposure to porphyrin compounds, calculated in the reference to the Fig 3.

**Treatment**	**Parameters of *S. aureus* growth**				
	**μ_{max}** [OD_{600}/h]	**Td** [hours]	**A_{max}** [OD_{600max}]	**Time of obtained stationary phase** [min]	***S. aureus* growth at the end of exponential phase** [%]
**Ga^{3+}MPIX**	0.15	4.65	0.621	480	77.43 ± 0.67 ***
**Ga^{3+}PPIX**	0.126	5.5	0.52	450	67.6 ± 0.8 ***
**PPIX**	0.282	2.45	0.75	390	101.9 ± 1.08
**$PPIXArg_2$**	0.282	2.45	0.76	390	104.5 ± 1.2
**Untreated**	0.354	1.95	0.77	390	100

*Legend: μ_{max}- maximum specific growth rate during exponential phase of bacterial growth; Td- Time of duplication, also known as generation time; A_{max}- maximal absorbance value with maximal bacterial density; Time of the obtained stationary phase - defined as the point of curve flattening; S. aureus growth at the end of exponential phase [%] at 240 min as the inflection point of the exponential growth curve calculated in the reference to control- untreated cells (100%). Significance at the respective p-values is marked with asterisks [***$p < 0.001$] with respect to untreated S. aureus 25923 cells.*

ROS detection and Quenchers in Ga^{3+}MPIX phototreatment

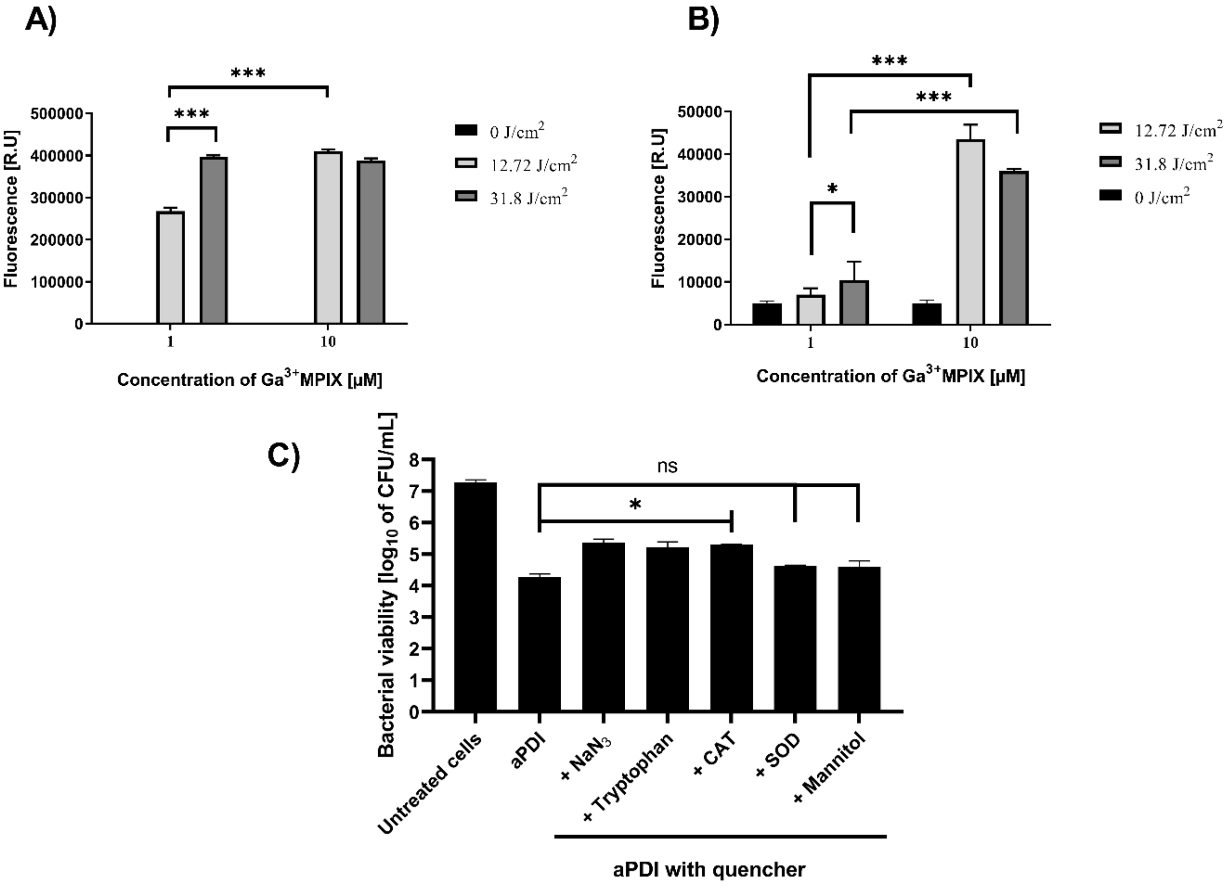

Figure S3. ROS detection and quenching in Ga^{3+}MPIX-mediated phototreatment. Cell-free suspensions of Ga^{3+}MPIX were incubated with ROS-detecting fluorescent probes: hydroxyl radical – HPF (3′-(p-hydroxyphenyl) Sigma–Aldrich, USA) (**A**) or singlet oxygen - Singlet Oxygen Sensor Green reagent (SOSG, Thermo Fisher Scientific, USA) (**B**) upon irradiation with LED light as indicated in the legend. Ga^{3+}MPIX was dissolved in PBS buffer, and then each fluorescent probe was added in darkness to a desired concentration of 5 µM. The fluorescence signal for both probes was detected immediately after green light irradiation at 12.72 J/cm^2 and 31.8 J/cm^2 doses, measured on an EnVision Multilabel Plate Reader at excitation/emission of 488/525 nm for SOGS and 490/515 nm for HPF. The values are the means of three separate experiments. (**C**) *S. aureus* 25923 (~10^7 CFU ml^{-1}) was incubated with 10 µM of **Ga^{3+}MPIX** exposed to LED-light at a dose of 25.44 J cm^{-2} (referred to as aPDI). A type II quencher (0.1 mM sodium azide, NaN_3), type I quencher (10 mM Mannitol, 1.35 units of superoxide dismutase - SOD, and 10 units of catalase - CAT), and a mixed type I/II quencher (0.1 mM tryptopxhan) were, respectively, pre-incubated with the mixture of PS and bacterial suspensions for 10 min prior to illumination. The experiment was conducted in three independent biological repetitions. Significance at the respective p values is marked with asterisks [* $p < 0.05$; *** $p < 0.001$].

Phototreatment of *S. aureus* with Ga^{3+}MPIX or Ga^{3+}PPIX reduced bacterial viability after additional wash-step

Table S3. Phototreatment of *S. aureus* strains with Ga^{3+}PPIX or Ga^{3+}MPIX with green LED light with wash-step.

**Strain**	**Mean reduction of survival (log_{10} CFU/mL)[1] ± SD**				
	**Ga^{3+}MPIX**		**Ga^{3+}PPIX**		**Light only**
	Light (+)	Light (-)	Light (+)	Light (-)	
**25923**	5.26 ± 0.83****	0.38 ± 0.54	2.25 ± 0.16**	0.615 ± 0.6	0.24 ± 0.26
**4046/13**	3.00 ± 0.13****	0.26 ± 0.19	1.26 ± 0.2****	0.25 ± 0.2	0.08 ± 0.01
**1814/06**	5.22 ± 0.39****	0.93 ± 0.13	3.54 ± 0.52****	0.28 ± 0.048	0.8 ± 0.09
**5N**	2.18 ± 0.68**	0.1± 0.39	0.84 ± 0.69	-0.11 ±0.05	0.14 ± 0.28

[1] phototreatment conditions: 10 µM Ga^{3+}PPIX or Ga^{3+}MPIX; green LED light 31.8 J/cm^2; log_{10} CFU/mL reduction was assessed with respect to non-treated cells, initial number of cell ~10^7 CFU/mL, cells were incubated with proper PS for 10 min, then washed once with PBS buffer and resuspended into fresh TSB medium. Light (+)- light dependent; Light (-)- light independent; light only- bacterial cells irradiated without any PS applied. Significance at the respective p-values is marked with asterisks [$p < 0.05$; **$p < 0.01$; ***$p < 0.001$, ****$p<0.0001$] with respect to Light only treated cells.*

Ga^{3+}MPIX-mediated photoinactivation of SCVs

A)

B)

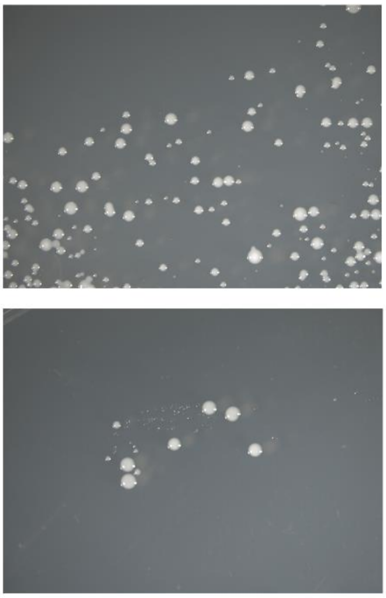

D)

E)

F)

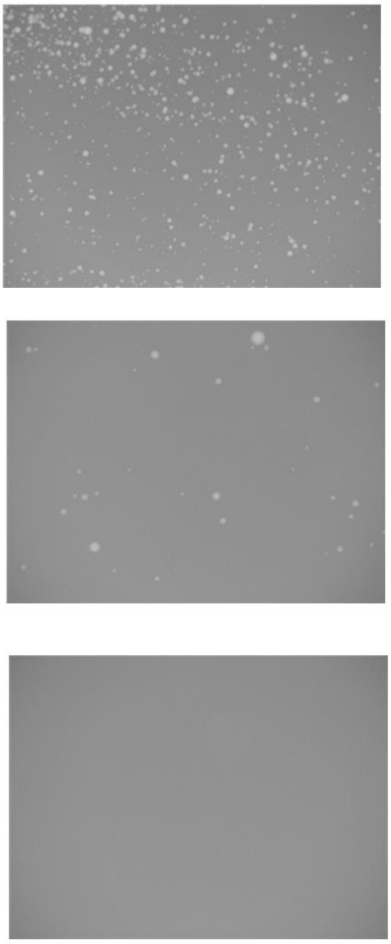

C)

Strain		Ga^{3+}MPIX concentration [µg/mL]	
		MIC[1]	SCV $_{[100\%]}$[2]
25923		0.54	34
4046/13		0.54	277
1814/06		17.3	138
5N		17.3	> 277
Newman	WT	2.16	138
	ΔI1rtA	1.08	69
	ΔHtsA	2.16	138
	ΔIsdD	2.16	138

Figure S4. A, B) SCVs generated after Ga^{3+}MPIX-mediated aPDI treatment. *S. aureus* 25923 bacteria plated on TSA plates (100 µl aliquots) survived after treatment (1 µM Ga^{3+}MPPIX, 25.4 J/cm^2) with (A) or without (B) iron in the culture medium. The microscopic photographs of plates were taken under a Leica MZ10 stereoscopic microscopy (Leica, Germany). **C) Effect of light-independent action of Ga^{3+}MPIX on several staphylococcal strains used in this study.** Overnight bacterial cultures were adjusted in fresh MHB medium to 0.5 McFarland, 100-fold diluted and finally transferred with or without PS to a 96-well plate. After 20 hours of incubation in 37°C, cells was plated to TSA agar plates and morphology of colonies were tested. [1] MIC referred as compound concentration when the inhibition of bacterial growth was significantly delayed; [2] SCV$_{[100\%]}$ - Concentration of Ga^{3+}MPIX in which only SCV morphology was detected (100%). **D, E, F) Efficiency of green light irradiation in SCVs photokilling after 20 hours exposure to Ga^{3+}MPIX [138 µg/mL].** *S. aureus* Newman WT was exposed to SCV$_{[100\%]}$ concentration of Ga^{3+}MPIX, then cells were illuminated with green light dosage of 12.72 J/cm^2 (E) or 25.44 J/cm^2 (F). Untreated cells were left for dark control (D). After that, cells were plated to TSA agar plates and microscopic photographs was taken under Leica MZ10 stereoscopic microscopy (Leica, Germany).

Impairment in the HrtA detoxification efflux pump promotes dark toxicity of Ga^{3+}MPIX

Table S4. ***S. aureus*** **Newman and its isogenic mutants growth under exposure to gallium- MPs, calculated in the reference to the Figure 8.**

S. aureus **isogenic mutant**	**Treatment**	Parameters of *Staphylococcus aureus* growth curve				
		**μ_{max}** [OD_{600}/h]	**Td** [hours]	**A_{max}** [OD_{600max}]	**Time to reach stationary phase** [min]	**Growth at the end of exponential phase** [%]
**WT**	**Untreated**	0.498	1.39	0.645	330	100
	**Ga^{3+}MPIX**	0.414	1.67	0.568	330	93 ± 1.44
	**Ga^{3+}PPIX**	0.39	1.7	0.45	390	74 ± 0.66
**ΔHrtA**	**Untreated**	0.52	1.32	0.654	360	100
	**Ga^{3+}MPIX**	0.36	1.9	0.586	480	**82 ± 2.3**
	**Ga^{3+}PPIX**	0.35	1.95	0.54	480	77 ± 1.5
**ΔIsdD**	**Untreated**	0.474	1.46	0.75	330	100
	**Ga^{3+}MPIX**	0.336	2.06	0.57	360	90 ± 2.93
	**Ga^{3+}PPIX**	0.37	1.86	0.49	360	73 ± 4.4
**ΔHtsA**	**Untreated**	0.47	1.48	0.7	330	100
	**Ga^{3+}MPIX**	0.43	1.62	0.63	390	90 ± 1.6
	**Ga^{3+}PPIX**	0.39	1.77	0.5	390	72 ± 1.5

Legend: μ_{max}- maximum specific growth rate during exponential phase of bacterial growth; Td- Time of duplication, also known as generation time; A_{max}- maximal absorbance value with maximal bacterial density; Time to reach stationary phase - defined as the point of curve flattening; S. aureus growth at the end of exponential phase [%] at 270 min as the inflection point of the exponential growth curve calculated in the reference to control- untreated cells (100%).

Ga^{3+}MPIX accumulation under confocal microscopic image

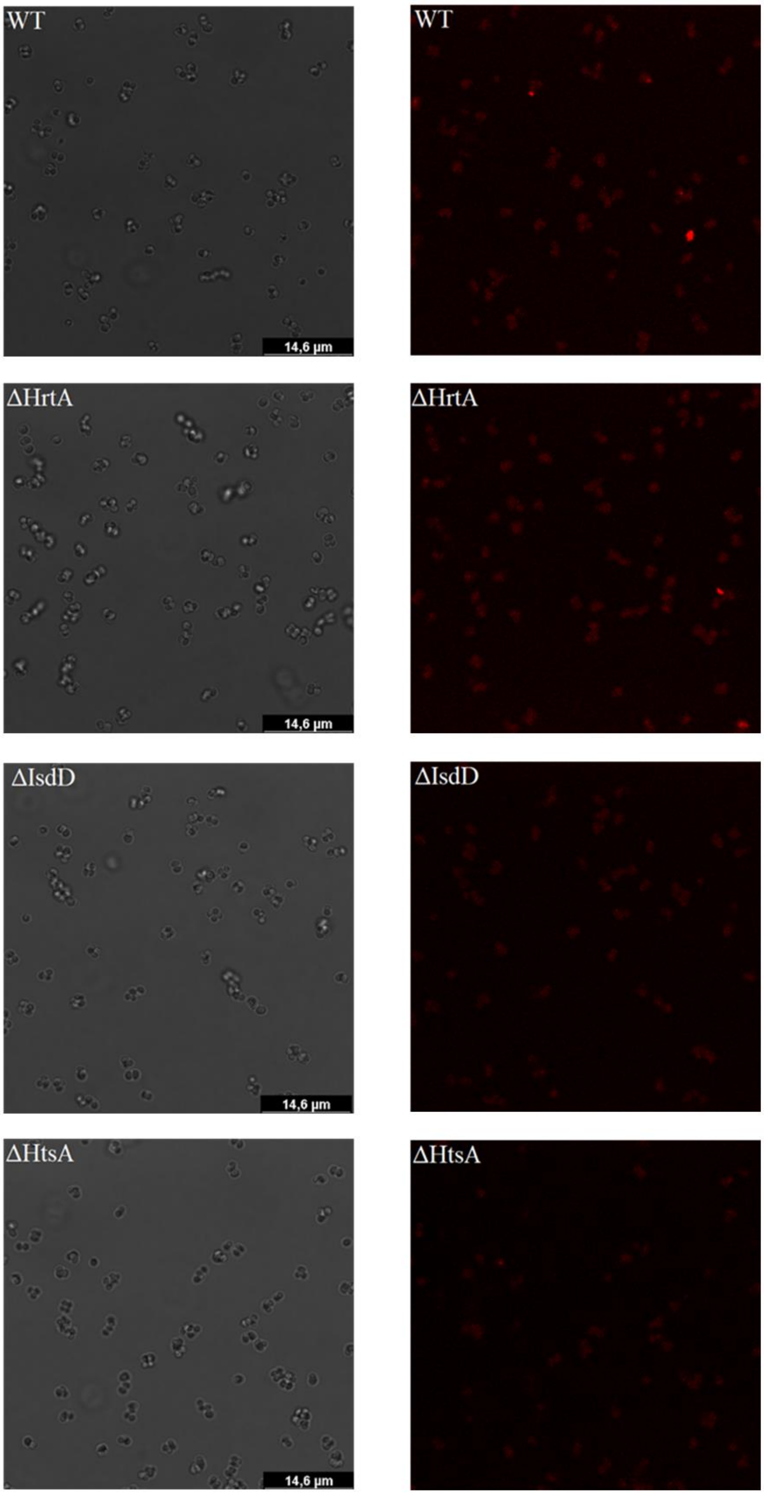

Figure S5. Ga^{3+}MPIX uptake in S. *aureus* Newman and its isogenic mutant (ΔHrtA, ΔIsdD and ΔHtsA) in the presence of iron. Overnight bacterial cultures were diluted and incubated with photosensitizer for 2 hours at 37°C with shaking. Then, washed once with PBS buffer. Specimens were imaged using a confocal laser scanning microscope Leica SP8X with a 63× oil immersion lens with excitation of 405 nm and fluorescence emission in 551-701 nm (Leica, Germany)

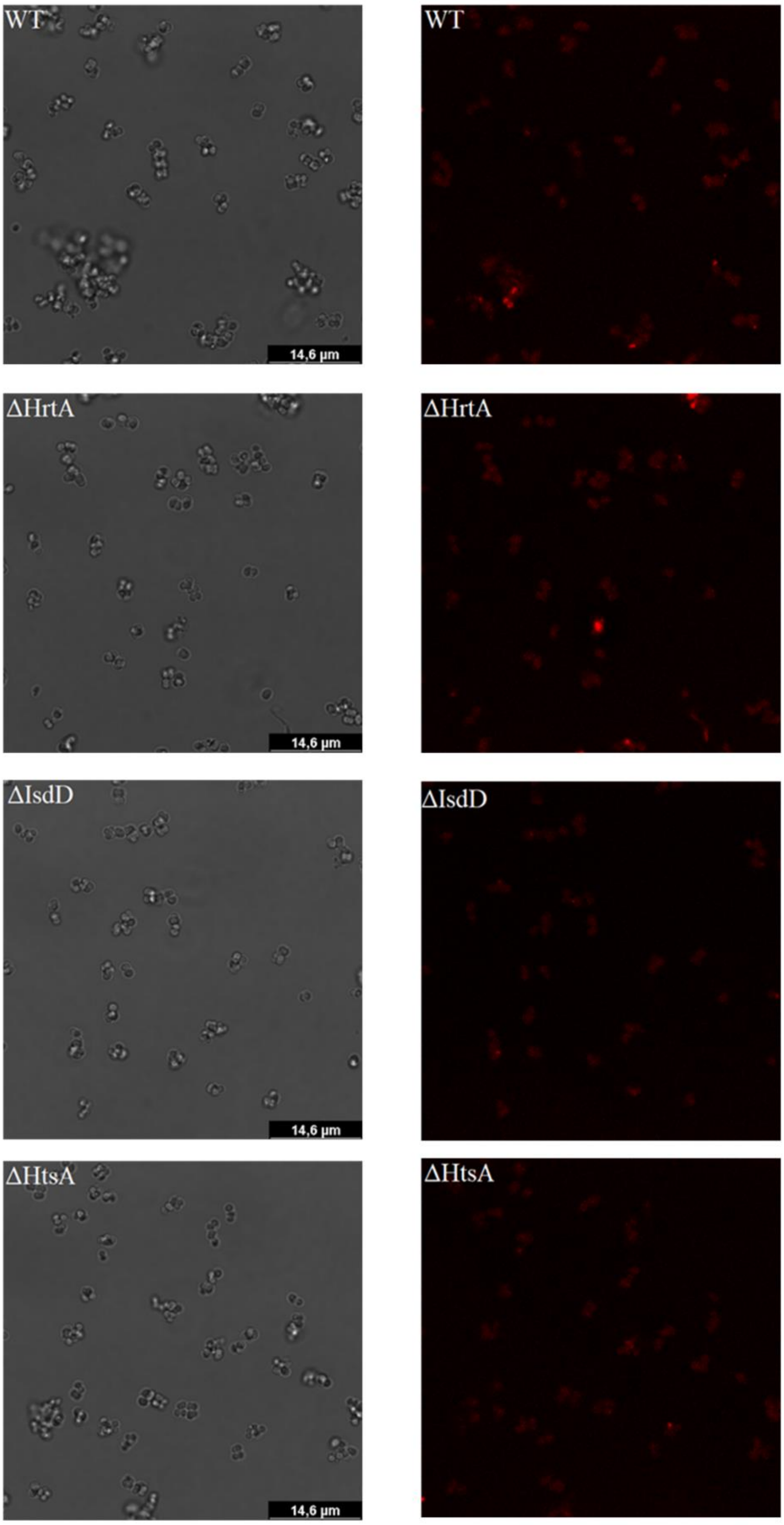

Figure S6. Ga^{3+}MPIX uptake in S. *aureus* Newman and its isogenic mutant (ΔHrtA, ΔIsdD and ΔHtsA) in the absence of iron. Overnight bacterial cultures were diluted and incubated with photosensitizer for 2 hours at 37°C with shaking. Then, washed once with PBS buffer. Specimens were imaged using a confocal laser scanning microscope Leica SP8X with a 63× oil immersion lens with excitation of 405 nm and fluorescence emission in 551-701 nm (Leica, Germany)

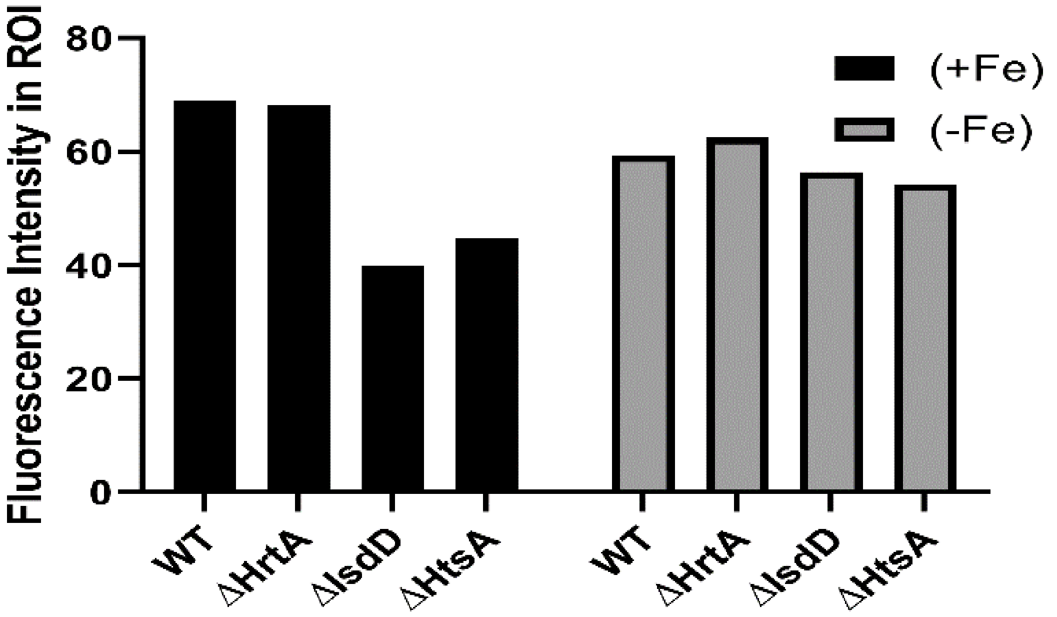

Figure S7. Maximal Fluorescence Intensity Amplitude in ROI measured on confocal microscopic images of uptake in S. *aureus* Newman and its isogenic mutant (ΔHrtA, ΔIsdD and ΔHtsA). Overnight bacterial cultures were diluted and incubated with Ga^{3+}MPIX for 2 hours at 37°C with shaking. Then, washed once with PBS buffer. The values represented on graph are the highest amplitudes of fluorescence measured in ROI length of 12.59 µmm.

$Ga3^{+}$MPIX does not promote extensive and prolonged cytotoxicity or phototoxicity against human keratinocytes

A)

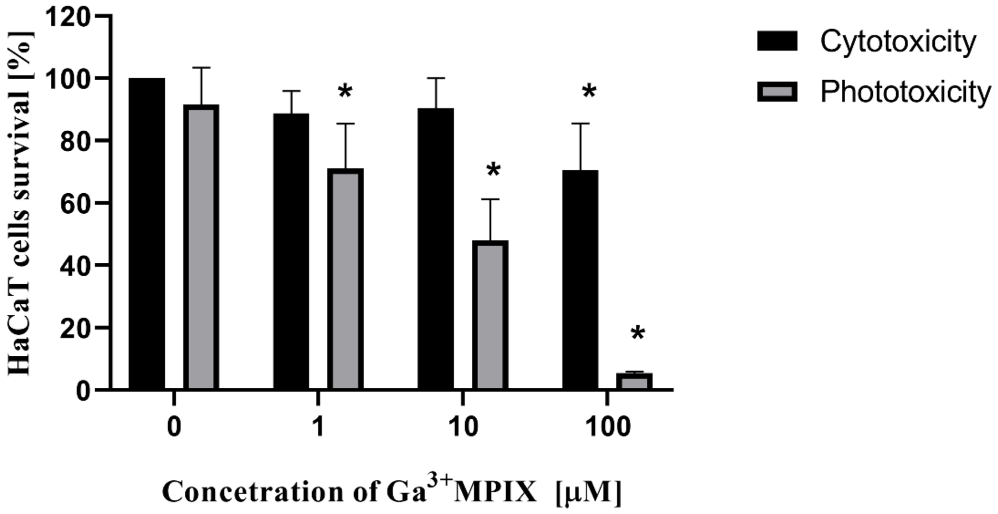

B)

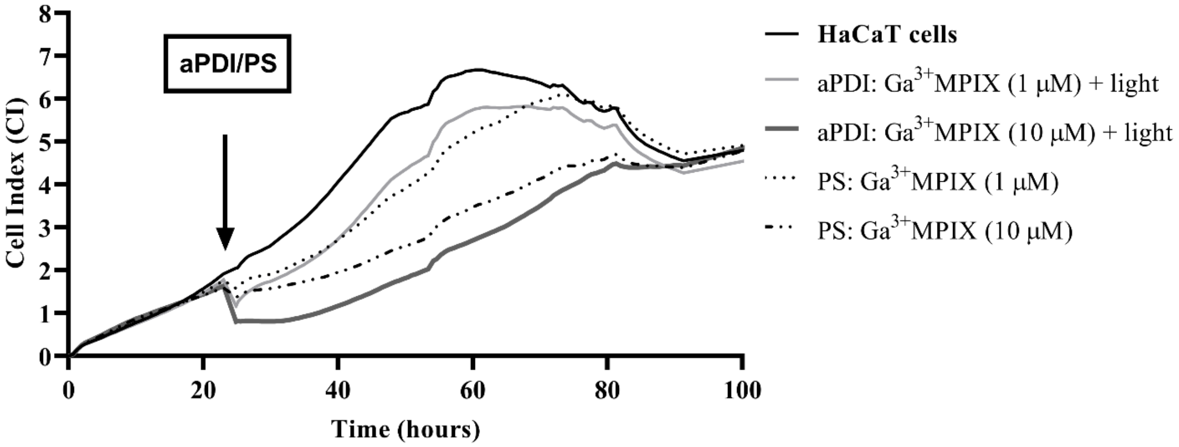

Figure S8. Effect of aPDI with Ga^{3+}MPIX on the HaCaT cell line model <u>with one-wash step</u>. A) MTT cell viability assay. HaCaT cells were exposed to various concentrations of Ga^{3+}MPIX. Control cells (0 µM) to which no test compound was added. After incubation with Ga^{3+}MPIX, cells were washed with PBS and either irradiated with green light (31.8 J/cm^2) represented by gray bars (Phototoxicity) or kept simultaneously in the dark (black bars for Cytotoxicity). Each result is the mean ± SD of the mean. Significance at the respective p values is marked with asterisks (∗ $p < 0.05$) for untreated cells (0 µM, Cytotoxicity). **B) Cell growth dynamics**. Cells were seeded at 10^4 cells/well and after obtaining a cell index (CI) 2, cells were treated with Ga^{3+}MPIX in the dark, incubated at 37 °C for 10 minutes. The samples were washed once then illuminated with a green light dose of 31.8 J/cm^2, while the HaCaT cells or PS-only treatment was allowed to incubate in the dark at room temperature. The CI (represented at the Y axis) was measured for each condition every 10 min. The x-axis shows the experiment duration in hours. The values presented are the average of the seven technical repetitions
